# The Rice B-Box Zinc Finger Gene Family: Genomic Identification, Characterization, Expression Profiling and Diurnal Analysis

**DOI:** 10.1371/journal.pone.0048242

**Published:** 2012-10-31

**Authors:** Jianyan Huang, Xiaobo Zhao, Xiaoyu Weng, Lei Wang, Weibo Xie

**Affiliations:** National Key Laboratory of Crop Genetic Improvement and National Center of Plant Gene Research (Wuhan), Huazhong Agricultural University, Wuhan, China; RIKEN Plant Science Center, Japan

## Abstract

**Background:**

The B-box (BBX) -containing proteins are a class of zinc finger proteins that contain one or two B-box domains and play important roles in plant growth and development. The *Arabidopsis BBX* gene family has recently been re-identified and renamed. However, there has not been a genome-wide survey of the rice *BBX (OsBBX)* gene family until now.

**Methodology/Principal Findings:**

In this study, we identified 30 rice *BBX* genes through a comprehensive bioinformatics analysis. Each gene was assigned a uniform nomenclature. We described the chromosome localizations, gene structures, protein domains, phylogenetic relationship, whole life-cycle expression profile and diurnal expression patterns of the *OsBBX* family members. Based on the phylogeny and domain constitution, the *OsBBX* gene family was classified into five subfamilies. The gene duplication analysis revealed that only chromosomal segmental duplication contributed to the expansion of the *OsBBX* gene family. The expression profile of the *OsBBX* genes was analyzed by Affymetrix GeneChip microarrays throughout the entire life-cycle of rice cultivar Zhenshan 97 (ZS97). In addition, microarray analysis was performed to obtain the expression patterns of these genes under light/dark conditions and after three phytohormone treatments. This analysis revealed that the expression patterns of the *OsBBX* genes could be classified into eight groups. Eight genes were regulated under the light/dark treatments, and eleven genes showed differential expression under at least one phytohormone treatment. Moreover, we verified the diurnal expression of the *OsBBX* genes using the data obtained from the Diurnal Project and qPCR analysis, and the results indicated that many of these genes had a diurnal expression pattern.

**Conclusions/Significance:**

The combination of the genome-wide identification and the expression and diurnal analysis of the *OsBBX* gene family should facilitate additional functional studies of the *OsBBX* genes.

## Introduction

Transcription factors (TFs) are key regulatory proteins that can activate or repress the transcription of their target genes and thus play critical roles in the life-cycle of higher plants [1]. The rice genome (*Oryza sativa* L. ssp. *japonica*) encodes 2384 putative TFs that are grouped into 63 different gene families [2]. The B-box (BBX) proteins are a class of zinc finger TFs that contained one or two B-box domains with specialized tertiary structures that are stabilized though the binding of Zn ions [3], [4]. In addition, the BBX proteins are likely to be involved in DNA binding or protein-protein interactions [5]–[8]. Typically, the plant BBX proteins contain one or two B-box domains near the amino terminus, and some have a CCT (CONSTANS, CO-like and TIMING Of CAB1) domain near the carboxy terminus [3]. Initially, many of the *Arabidopsis BBX* genes were named as *CO-Like* (*COL*) because *CO* (*CONSTANS*) was the first identified *BBX* gene in *Arabidopsis*. Khanna et al. established a uniform terminology for the genomic identification of the 32 BBX members in *Arabidopsis* and renamed them as *AtBBX* genes [3], [9], [10].

The best-known AtBBX protein, CO, contains two B-box and one CCT domains, and plays an important role in the photoperiod regulation of flowering. *CO* promotes flowering under long-day (LD) conditions but has no effect on the flowering time under short-day (SD) conditions [5]. Unlike *CO*, the altered expression of *BBX2* (*COL1*) and *BBX3* (*COL2*) has little effect on the flowering time but the over-expression of *BBX2* can shorten the period of two distinct circadian rhythms [11]. *BBX4* (*COL3*) is a positive regulator of photomorphogenesis and promotes root growth [12]. BBX6 (COL5) works as an SD-specific inducer of flowering by promoting the expression of *FT* [13]. BBX7 (COL9) functions as an LD-specific repressor of flowering by reducing the expression of *CO* and *FT* [14]. A number of AtBBXs that only have two B-box domains have also been identified: BBX18 (DBB1a), BBX19 (DBB1b), BBX24 (STO) and BBX25 (STH1) are negative regulators of light signaling [3], [9], [10], [15]–[17], whereas BBX21 (STH2) and BBX22 (LZF1/STH3) are positive regulators [18]–[20]. BBX32, which contains only a single B-box, is also a modulator of light signaling [21].

In rice, only three *BBX* genes have been identified to date. The rice CO ortholog, Hd1, is a typical BBX protein that contains two B-box and one CCT domains. *Hd1* has a parallel function in the regulation of flowering time, but promotes flowering under SD rather than LD conditions [22]. OsCO3 contains a single B-box and a CCT domain and modulates the photoperiodic flowering in rice [23]. The overexpression of *OsCO3* in rice causes late flowering under SD conditions but does not affect the flowering time in *Arabidopsis*. OsCOL4 functions as a constitutive repressor of flowering under SD and LD conditions [24], [25].

Hence, it is timely to comprehensive analyze the *BBX* family in rice and provide a uniform nomenclature for this class of proteins. In this study, 30 rice *BBX* genes *(OsBBXs)* were identified through a genome-wide survey. The chromosomal location and phylogenetic relationship of the OsBBXs were performed. The expression profile of the *OsBBX* genes in the rice life-cycle was analyzed using Affymetrix GeneChip microarrays and confirmed by qPCR. The diurnal expressions of the *OsBBX* genes were analyzed through the Diurnal Project and qPCR. In addition, the expression analysis of the rice *BBX* genes under light/dark conditions and after phytohormone (NAA, KT and GA_3_) treatments was performed. To our knowledge, this is the first report that focuses on the family-level identification of the *OsBBX* genes and analyzes their expression patterns. These results therefore provide a solid base for future functional genomic studies on the *BBX* gene family in rice.

## Materials and Methods

### Sequence retrieval and gene family member identification

The BLAST search tools BLASTP and TBLASTN [26] were used to identify the putative OsBBX with the AtBBX22 (AT1G78600) protein sequence and the conserved sequence of B-box domain as queries against four databases: the MSU Rice Genome Annotation Project Database (Release 7 of MSU RGAP) (http://rice.plantbiology.msu.edu/) [27], the Rice Annotation Project Database (RAP-DB, Rice genome IRGSP/RAP build 5) (http://rapdb.dna.affrc.go.jp/) [28], [29], the National Center for Biotechnology Information (NCBI) (http://www.ncbi.nlm.nih.gov/) and the Knowledge-Based Oryza Molecular Biological Encyclopedia (KOME) (http://cdna01.dna.affrc.go.jp/cDNA/) [30]. The BLASTP and TBLASTN search parameters of these databases were set as follows: expect value <10 and 500 maximum target sequences. The domain search and putative function search with the key word B-box were used to search against the MSU RGAP. The SMART (http://smart.embl-heidelberg.de/) [31], [32], Pfam (http://pfam.sanger.ac.uk/) [33] and InterProScan (http://www.ebi.ac.uk/Tools/pfa/iprscan/) searches were used to confirm the presence of the B-box domain and to predict the domain composition of each OsBBX protein. Information about the transcript, chromosomal position, full-length cDNA and characteristics of the proteins were obtained from MSU RGAP. The gene structure of each gene was displayed using the Gene Structure Display Server (http://gsds.cbi.pku.edu.cn/) [34]. The available mutant lines for some of the *OsBBX* genes were identified in the RiceGE database (http://signal.salk.edu/cgi-bin/RiceGE).

### Determining chromosomal localization and gene duplication

The distribution of the *OsBBX* genes on the chromosomes was drawn using the MapChart software [35] according to the physical positions of the *OsBBX* genes and modified manually with annotation. The *OsBBX* genes that are present on duplicated chromosomal segments were identified by segmental genome duplication of rice, which is available at MSU RGAP (http://rice.plantbiology.msu.edu/segmental_dup/index.shtml). The maximum distance permitted between the collinear gene pairs was 500 kb. The *OsBBX* genes that were separated by a maximum of five genes were considered as tandemly duplicated genes.

### Phylogenetic analysis and sequence alignment

Multiple sequence alignments of the amino acid sequences were generated using ClustalW in MEGA 5.05 with the default parameters [36]. The obtained sequence alignments were used as the input for the neighbor-joining algorithm in MEGA 5.05 to construct the phylogenetic trees of these sequences. Bootstrap analysis was performed using 1000 replicates. The phylogenetic tree was also displayed and annotated using MEGA 5.05. The multiple sequence alignment that was generated using ClustalW was modified manually, and the conserved sequences were annotated according to the results obtained from the SMART and Pfam searches. The logos of the conserved motifs were generated by submitting the sequence alignments to WebLogo [37].

### Expression profile analysis, phytohormone and light/dark treatments

The original expression data for the *OsBBX* genes were extracted from the CREP database, which was developed by our lab (GSE19024) [38]. Thirteen vegetative and eleven reproductive samples of different developmental stages that cover the whole life-cycle of rice cultivar Zhenshan 97 (ZS97) were used for the *OsBBX* expression profile analysis that was performed in this study (Table S1). The unique probe set for each *OsBBX* gene was chosen according to the criterion mentioned in a previous study [39].

The quantile normalization and summarization of the origin microarray data were performed following the method described by Wang et al [38]. The average signal value of the replicates for each probe set was used for the analysis. The expression value of each probe set was logarithmized and transformed by Z-score. The relative expression values (Z-score) were used for hierarchical cluster analysis using the method of complete linkage based on the Euclidean distances by R-2.9.0 (http://www.r-project.org/). For phytohormone and light/dark treatments, the expression values was logarithmized and used for hierarchical cluster analysis by complete linkage method.

The samples used for the qPCR verification of the whole life-cycle and light/dark treatments were the same samples that were used for the Affymetrix microarrays. In the phytohormone treatments, germinating ZS97 seeds were sown in the greenhouse. The seedlings were treated with 100 µM gibberellin (GA_3_), auxin (NAA) or cytokinin (KT) at the trefoil stage, and samples that were collected after treatment for 5, 15, 30 and 60 min were pooled for each hormone treatment.

### qPCR analysis

The TRIzol reagent (Invitrogen, CA, USA) was used to extract the total RNA according to the manufacturer's instructions. A total of 3 µg RNA was treated with DNase I (Invitrogen, CA, USA) to eliminate any contaminating DNA. The first-strand cDNA was reverse transcribed from the total RNA using the M-MLV reverse transcriptase (Invitrogen, CA, USA). The primers used for the qPCR are listed in Table S2. The rice *Ubiquitin* (*LOC_Os03g13170*) gene was used as the internal control. The qPCR was performed in a total volume of 25 µl containing 2 µl of the reverse-transcribed product cDNA, 0.2 µM of each primer, 12.5 µl of SYBR Premix Ex Taq and 0.5 µl of ROX Reference Dye II (Takara, Japan) using the Applied Biosystems 7500 Real-Time PCR System. The data were analyzed using the relative quantification method [40].

### Diurnal expression patterns and *cis*-element analysis

A preliminary analysis of the diurnal expression pattern of the *OsBBX* genes was obtained from the Diurnal Project (http://diurnal.cgrb.oregonstate.edu/) [41], [42]. Two treatments were chosen for the analysis: light/thermocycles (LDHC): exposed to 12 h light (L), and 12 h dark (D) cycles with a high day temperature of 31°C and a low night temperature of 20°C. Plants in the LLHH (LDHC) group were exposed to the LDHC treatment followed by a two-day exposure to continuous light at a high temperature (31°C). qPCR was used to validate the diurnal expression patterns of several *OsBBX* genes. The samples used for the qPCR analysis were treated as in a previous study [43]. Young leaves were harvested from three different plants as three biological replications for each treatment. The samples were collected in 4 h intervals, starting at 8:30 for a total of 24 h. The qPCR was performed following the protocol that was described above.

To identify the putative *cis*-regulatory elements that exist in the *OsBBX* genes, the genomic sequences that are 1000 bp upstream from the translational start codons were used to search the PlantCARE database (http://bioinformatics.psb.ugent.be/webtools/plantcare/html/) [44].

## Results

### The *BBX* gene family members in rice

To identify the candidate *BBX* genes in the rice genome, we searched four databases: MSU RGAP, RAP-DB, NCBI and KOME. After removing the repeated sequences and different transcripts of the same gene, we identified 60 putative *OsBBX* genes. A domain search (PF00643) and putative function search of the MSU RGAP database only identified 30 putative *OsBBX* genes. The protein sequences of these genes were used for SMART, Pfam and InterProScan searches to confirm the presence of the B-box domain in each sequence. A total of 30 genes were confirmed to encode BBX proteins in rice. The B-box TFs that belong to the previous CO-like family also have been identified and reported in PlantTFDB (http://planttfdb.cbi.edu.cn/) (15 distinct B-box TFs and six repeated B-box TFs) [45], PlnTFDB (http://plntfdb.bio.uni-potsdam.de/v3.0/) (17 distinct B-box TFs and six repeated B-box TFs) [46], GramineaeTFDB (http://gramineaetfdb.psc.riken.jp/) (25 members with two incorrectly assigned members) [47] and Grassius (http://grassius.org/) (eight members) databases [48], [49]. The results of this study were compared with these databases and no new member was identified. For convenience, the 30 *OsBBX* genes were named as *OsBBX1* to *OsBBX30* according to their positions on the rice chromosomes. The detailed information on the RGAP Loci, RAP Os IDs, full-length cDNAs, transcripts, chromosomal positions, characteristics of the corresponding proteins, mutant lines and the original names of the 30 *OsBBX* genes are listed in Table 1. The gene structure of each *OsBBX* gene is shown in Figure S1.

**Table 1:** The detailed information of *OsBBX* members.

Name	RGAP LOC	RAP Os ID	Originalname	flcDNA	Genomic position	Gene orientation	CDS length	Protein length	Mutant lines
OsBBX1	LOC_Os01g10580	Os01g0202500	OsDBB3c	AK242176	5638769–5640495	Upward	1074	357	2C-30193, 2A-20608
OsBBX2	LOC_Os02g07930	Os02g0176000	N/A	AK241477	4149767–4151241	Downward	669	222	2A-50063, 2A-70002
OsBBX3	LOC_Os02g08150	Os02g0178100	OsF	AK109630	4315272–4316958	Upward	1050	349	N/A
OsBBX4	LOC_Os02g39360	Os02g0606200	OsSTH	AK104293, AK061044, CT830238, AB001885	23753345–23754688	Upward	816	271	3A-06908, 1B-23515, RdSpm2065A_3.1, K-05221
OsBBX5	LOC_Os02g39710	Os02g0610500	OsCOL4/OsD	AK100097, AK058536	23983881–23985424	Upward	999	332	GSNU_Ds48, 1C-02709
OsBBX6	LOC_Os02g43170	Os02g0646200	N/A	AK071507, CT832305	26021916–26023624	Upward	810	269	1C-09019, 4A-50785, 2D-21119, 3C-00699
OsBBX7	LOC_Os02g49230	Os02g0724000	OsN	CT832281, CT832280, CT832282, CT832279, AK071630, AB001888	30088390–30093805	Upward	1443	480	1B-01905, 03Z11BI26, T28979T, T29560T, K-02893, 3A-03271, H0027_1_204_1A
OsBBX8	LOC_Os02g49880	Os02g0731700	OsK	AK120314, AK072346	30466919–30469904	Downward	1359	452	GSNU_Ds56, DAI1F05
OsBBX9	LOC_Os03g22770	Os03g0351100	OsP	AK101822, AK099864, AB001882	13151678–13154262	Upward	1212	403	GSNU_Ds123
OsBBX10	LOC_Os03g50310	Os03g0711100	OsJ	AK120563	28680012–28682770	Upward	1266	421	1A-21121, SAO6E12,
OsBBX11	LOC_Os04g41560	Os04g0493000	OsSTO	AK061664, AB001883	24462763–24464258	Downward	774	257	3A-52431, 3D-01977, 1A-23305
OsBBX12	LOC_Os04g42020	Os04g0497700	OsC	AK121024, AK059443	24704844–24706348	Upward	1002	333	N/A
OsBBX13	LOC_Os04g45690	Os04g0540200	N/A	AK061333, CT830169	26842136–26844370	Upward	753	250	2A-50066, 1D-04450
OsBBX14	LOC_Os05g11510	Os05g0204600	OsDBB3b	AK106865	6514687–6517221	Upward	1137	378	T00477T, 05NPBLD37 04Z11RN03
OsBBX15	LOC_Os06g01340	N/A	N/A	N/A	208204–209107	Upward	672	223	1C-06536, M0022530 RDs501_5.2, RDs686_5.1
OsBBX16	LOC_Os06g05890	Os06g0152200	OsDBB3a	AK072231, AK104083, AB001886	2694461–2698504	Upward	1083	360	3B-00658, 3A-07296 1A-06932
OsBBX17	LOC_Os06g15330	Os06g0264200	OsL	AK287836, AK241249	8703832–8705950	Upward	1347	448	DAK3H04
OsBBX18	LOC_Os06g16370	Os06g0275000	Hd1/OsA	AK241018, AB041838	9335360–9337644	Upward	1188	395	T09919T, T31488T, T40187T, T32475T, T09921T, T17365T, T34409T, T10771T
OsBBX19	LOC_Os06g19444	Os06g0298200	OsM	AK072192, AK121630, AK098865, CT833045	11069109–11075722	Downward	1227	408	5A-00300, M0065138, NE2301
OsBBX20	LOC_Os06g44450	Os06g0654900	OsE	AK243265, CT837522	26842090–26843779	Downward	1116	371	4A-04189, 04Z11IK06
OsBBX21	LOC_Os06g45040	Os06g0661200	N/A	N/A	27252386–27254042	Upward	825	274	3B-00549, 1A-15201, 05NPBMT81
OsBBX22	LOC_Os06g49880	Os06g0713000	N/A	AK105957, CT833308, CT831615, AB001884	30195099–30196607	Upward	927	308	2B-20503, 3A-51129
OsBBX23	LOC_Os07g47140	Os07g0667300	N/A	AK120101	28183868–28186917	Downward	1143	380	04Z11HI63, 05NPBNR43, FL047220
OsBBX24	LOC_Os08g08120	Os08g0178800	N/A	AK287608, AK063025	4609503–4611921	Downward	852	283	NE1871
OsBBX25	LOC_Os08g15050	Os08g0249000	OsG	AK109938, CT833687	9097577–9098884	Upward	912	303	N/A
OsBBX26	LOC_Os08g42440	Os08g0536300	OsO	AK099722	26790110–26794467	Downward	1467	488	2C-60235, 1D-05321, 2C-20399, 3A-51217, 2D-30775, 3A-03017, 05NPBNP03, 05NPBNZ01
OsBBX27	LOC_Os09g06464	Os09g0240200	OsCO3/OsB	AK241570, CT835894, AB001887	3047020–3063571	Downward	1008	335	1C-03305, 1C-03306
OsBBX28	LOC_Os09g33550	Os09g0509700	N/A	AK120589	19783043–19786296	Downward	1416	471	T28637T, NE4523_0_503_1A, T09425T, T09366T, FL007537
OsBBX29	LOC_Os09g35880	Os09g0527900	OsDBB1	AK058794, AK122172, CT828272	20645916–20649512	Downward	636	211	3C-00717, 4A-01958
OsBBX30	LOC_Os12g10660	Os12g0209200	N/A	AK067820	5698772–5701407	Upward	633	210	2C-50216

### Protein sequence and phylogenetic analysis of the OsBBX family

**Figure 1:**
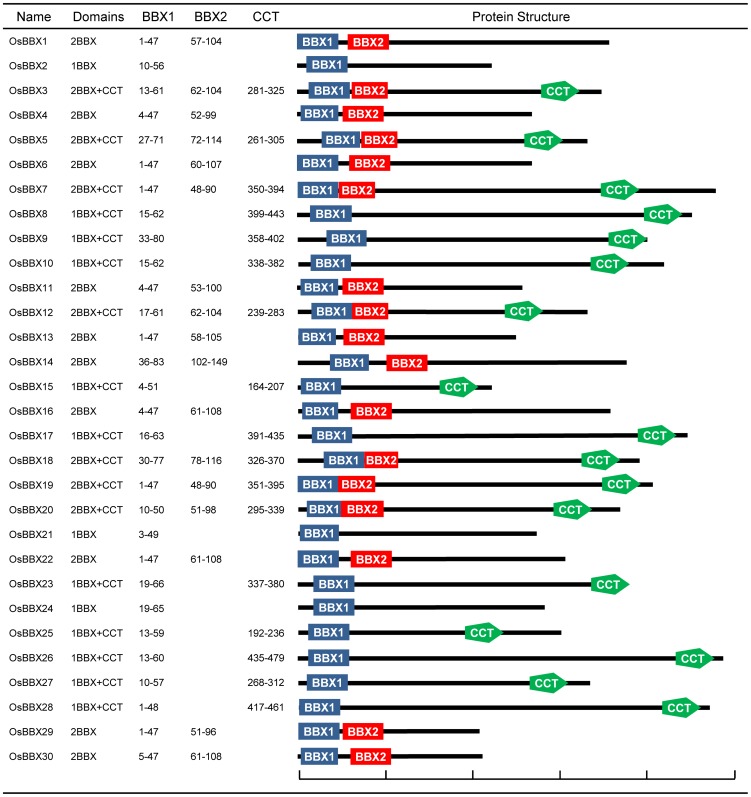
Structures of the OsBBX proteins. The name of each corresponding protein is shown on the left. The position of each domain is indicated in the figure. The length and order of the domains represent their actual location within each protein. The scale bar represents 100 amino acids.

**Figure 2:**
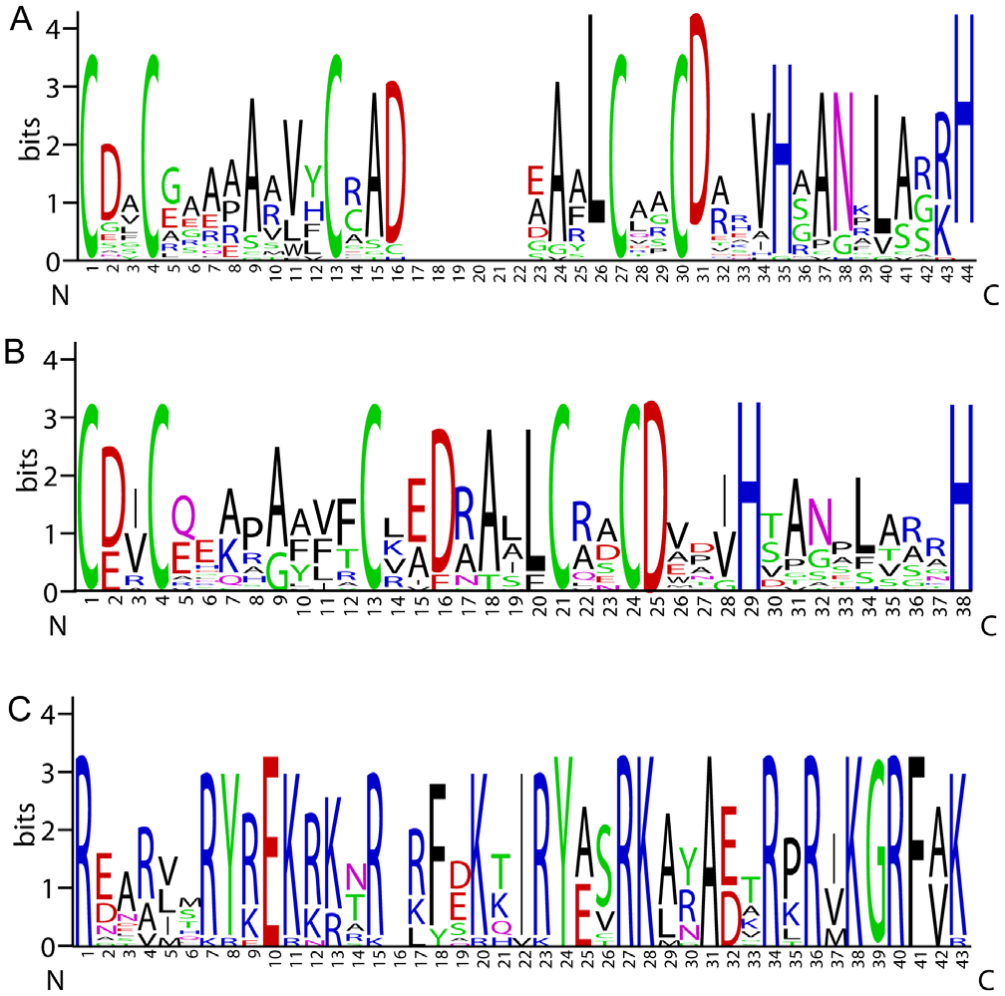
The conserved domains in the OsBBX proteins. Logos of the protein alignment of the B-box 1 (A), B-box 2 (B) and CCT domain (C) are shown. The x-axis represents the conserved sequences of the domain. Conservation of each residue across all proteins is indicated by the height of each letter. The y-axis is a scale of the relative entropy, which reflects the conservation rate of each amino acid.

**Figure 3:**
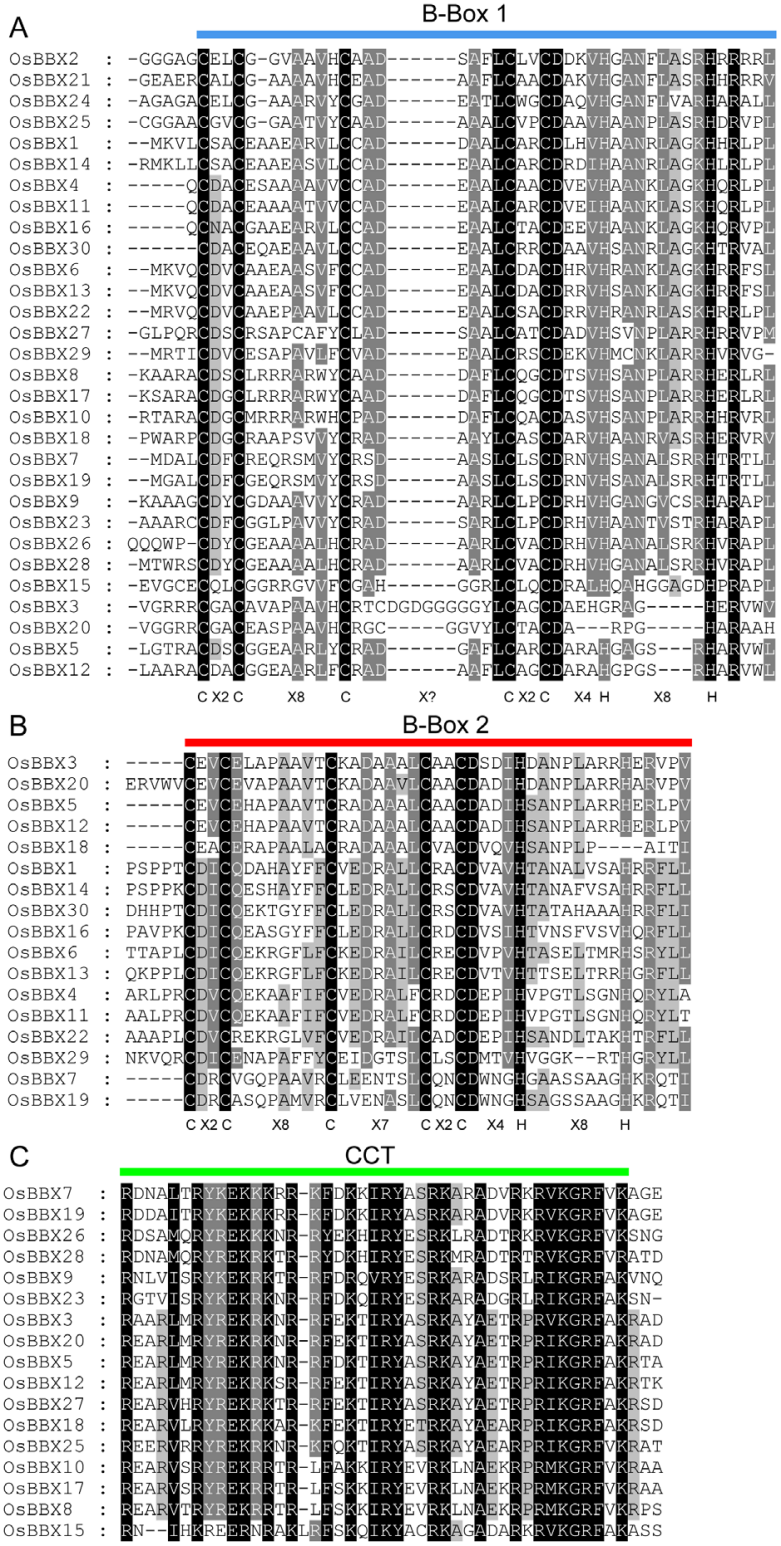
Multiple sequence alignments of the domains of the OsBBXs. Multiple sequence alignments of the B-box 1 (A), B-box 2 (B) and CCT (C) domains are shown. The identical amino acid, conserved amino acid and blocks of similar amino acid residues are shaded in black, charcoal gray and gray, respectively.

The OsBBX proteins vary widely in size, ranging from 210 to 488 amino acids in length. We identified seven OsBBXs that contain two B-box domains and a conserved CCT domain. Ten OsBBXs contained one B-box and one CCT domain. Three OsBBXs contained only one B-box domain, and 10 contained two B-box domains (Figure 1). The protein sequence alignment showed that the B-box 1 and B-box 2 domains of the OsBBXs have similar conserved sequences. The conserved sequence of B-box 1 is CDACGAAAAAVYCRADEAALCAACDAEVHAANKLARRH and the B-box 2 is CDVCEEAPAAVFCKEDRALLCRACDVDVHSANSLAARH. In addition, the Zinc finger domain in B-box 1 is in the form of C-X_2_-C-X_8_-C-X_?_-C-X_2_-C-X_4_-H-X_8_-H, whereas in B-box 2 is in the form of C-X_2_-C-X_8_-C-X_7_-C-X_2_-C-X_4_-H-X_8_-H. The CCT domain among the OsBBXs proteins is highly conserved. The logos of these domains in the OsBBXs proteins are shown in Figure 2, and their locations are illustrated in Figure 3.

**Figure 4:**
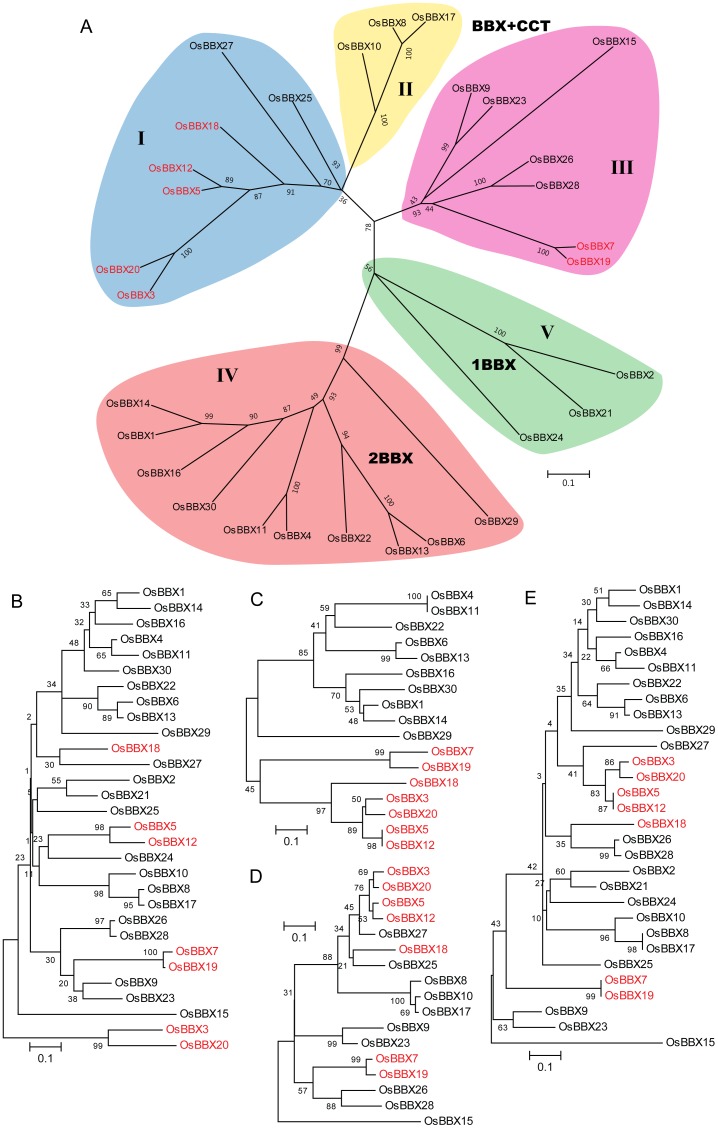
Phylogenetic analysis of the OsBBX family. The trees shown are based on the alignments of the protein sequences of the full length (A), the B-box 1 domain (B), B-box 2 domain (C), CCT domain (D) and concatenated B-box and CCT domains (E). The bootstrap values are indicated at each node. The scale bar represents 0.1 amino acid substitutions per site. The members marked in red contain two B-box and one CCT domains.

To examine in detail the phylogenetic relationship and functional divergence of the OsBBX members, the aligned 30 OsBBX protein sequences were used to construct an unrooted phylogenetic tree (Figure 4A). We also built the phylogenic tree including AtBBXs and OsBBXs as well as BBXs from maize, poplar and *sorghum bicolor* (Figure S4). Moreover, the aligned B-box 1 (Figure 4B), B-box 2 (Figure 4C), CCT (Figure 4D) and concatenated B-box and CCT (Figure 4E) domain sequences were also used for the phylogenetic analysis. The phylogenetic tree of OsBBX was further divided into 5 subfamilies based on our analysis and the previous study by Griffiths et al [24] (Figure 4). The members of subfamilies I, II and III are the OsBBXs that contain both B-box and CCT domains. We were unable to classify the OsBBXs that contain one B-box domain with a CCT domain and two B-box domains with a CCT domain into different subfamilies, which is different from the analysis of the phylogenetic tree of the AtBBXs. Rice, however, possesses two classes of BBXs that were not found in *Arabidopsis*. One class is in the subfamily I members, which contain a single B-box domain (OsBBX25 and OsBBX27). These two BBXs proteins evolved through the internal deletion of the second B-box domain [24]. The other class is in the subfamily III members, which includes five OsBBXs that have one B-box and one CCT domain, except that OsBBX7 and OsBBX19 possess the second B-box domains, which is different from the other members that contain two B-box domains. Subfamily II consists of three OsBBXs proteins with one B-box and one CCT domain. Subfamily IV contains 10 OsBBXs with two B-box domains, and subfamily V includes three OsBBXs with only one B-box domain.

### Chromosomal localization and gene duplication

**Figure 5:**
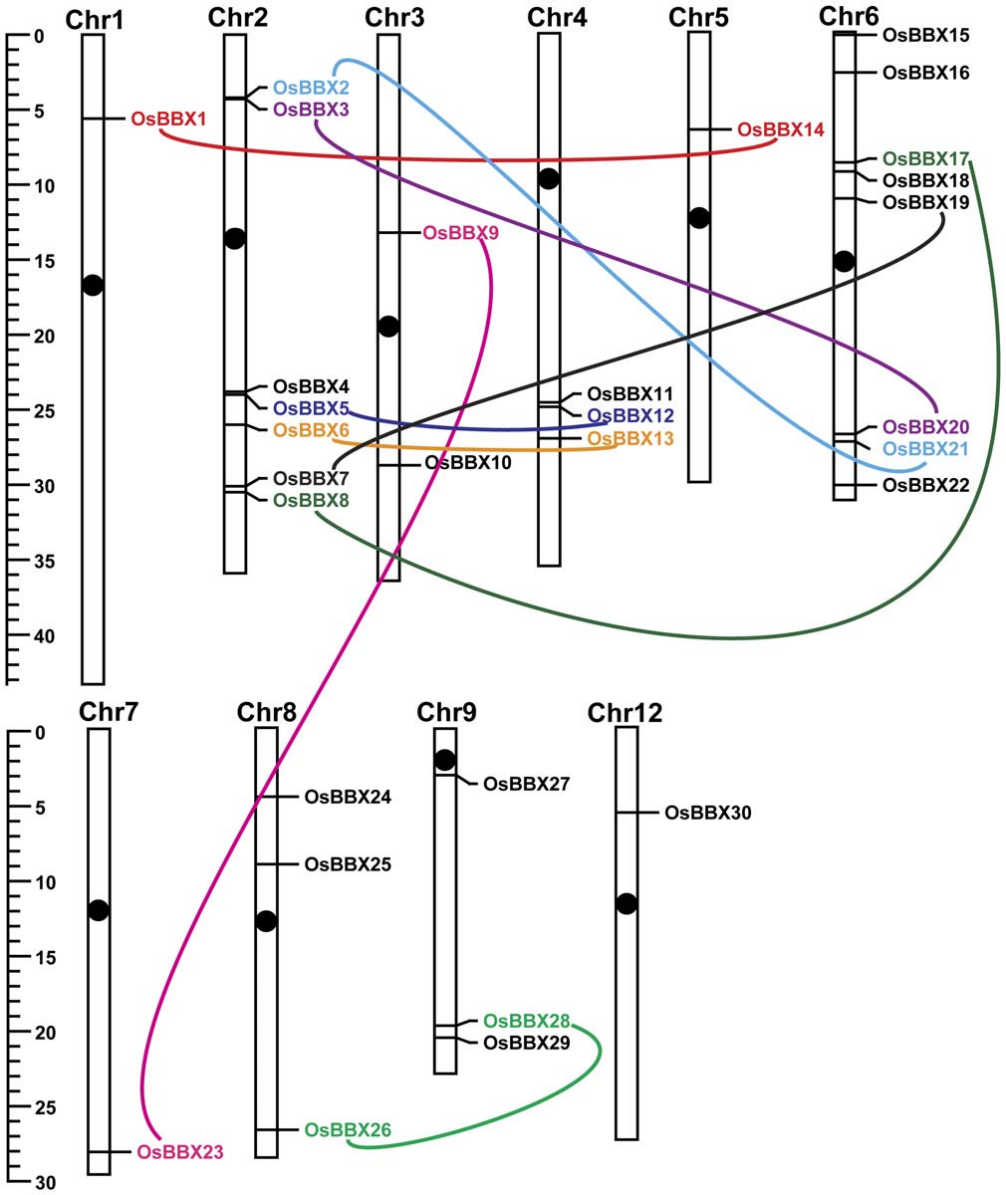
Distribution of the *OsBBX* genes on the rice chromosomes. The black ovals on the chromosomes indicate the position of the centromeres. The scale on the left is in megabases (Mb). The chromosome numbers are indicated at the top of each bar. The segmental duplicated genes are indicated in a different color and are connected by lines.

To determine the genomic distribution and the gene duplication of the *BBX* genes in rice, the approximate positions of the *OsBBX* genes were marked on the physical map of the rice chromosomes. According to the genomic localization, the 30 *OsBBX*s are distributed among all chromosomes except chromosomes 10 and 11. Only one *OsBBX* gene is found on chromosomes 1, 5, 7 and 12; seven genes are located on chromosome 2; two genes are localized on chromosome 3; three genes are found on chromosomes 4, 8 and 9, and eight genes are located on chromosome 6. The distribution of the *OsBBX* genes on the rice chromosomes is shown in Figure 5. Both the segmental and tandem duplications of the *OsBBX* gene family were analyzed. However, none of the *OsBBX*s are arranged in tandem. 18 *OsBBX*s are located in the duplicated segmental regions of rice chromosomes mapped by MSU RAGP (Figure 5).

### Expression profile of the *OsBBX* genes in the whole life-cycle of rice

**Figure 6:**
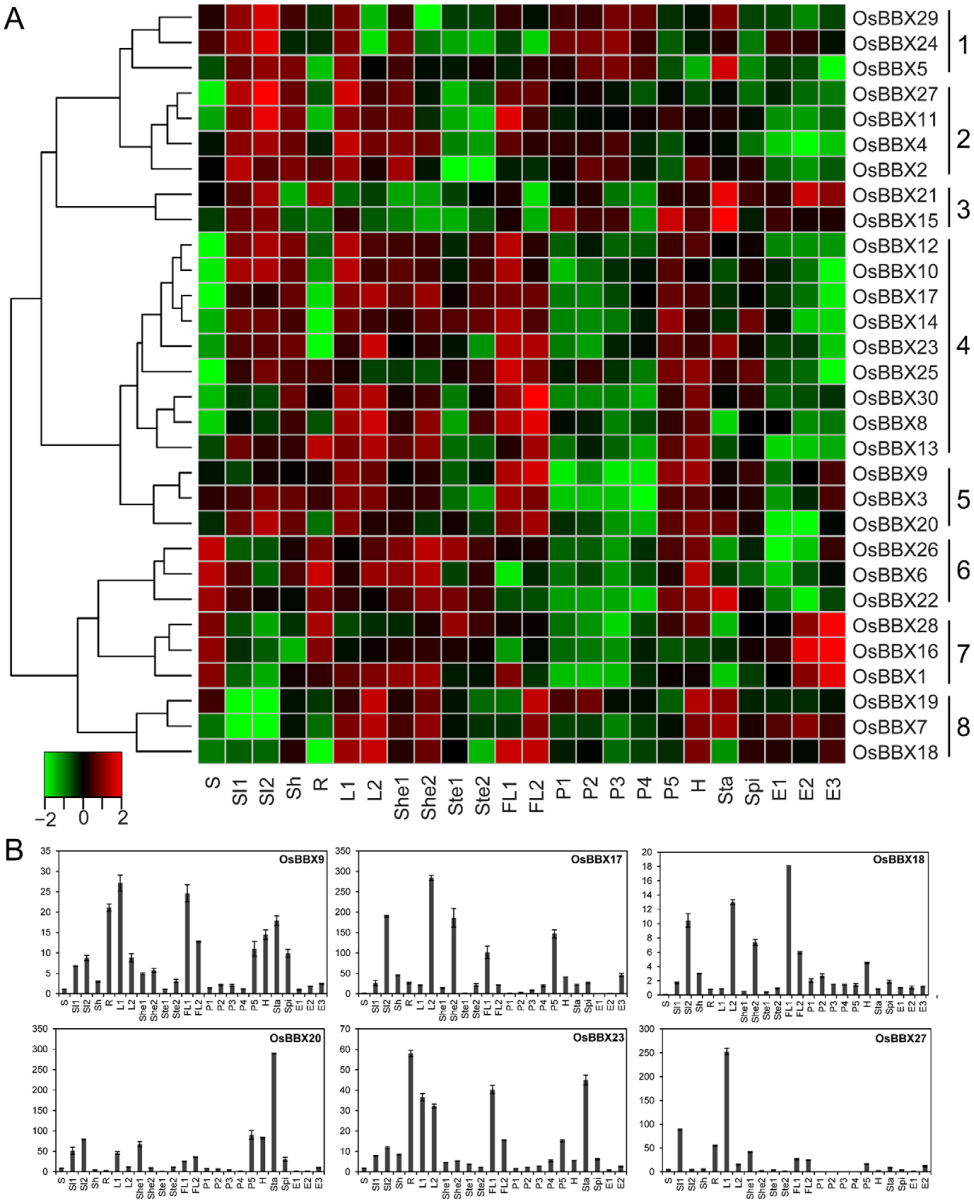
Expression profile of the *OsBBX* genes in the whole life-cycle of ZS97. (A) Hierarchical cluster displaying the expression profiles of the 30 *OsBBX* genes. The tissues used for the expression profiling are indicated at the bottom of each column. A cluster dendrogram is shown on the left. The color key at the bottom represents the Z-score values transformed from log_2_-based expression values. The qPCR verification results of some of the *OsBBX* genes are shown (B). The x-axis of the qPCR results shows samples as listed in Table S1. The y-axis is the relative expression level of each gene compared to its expression level in the Endosperm 1 tissue. The error bars indicate the standard deviations of the independent biological replicates.

To analyze the expression profile of the *OsBBX* genes on a global scale, including a wide range of tissues at different developmental stages, we extracted the expression data of the *OsBBX* genes from the CREP database. All 30 *OsBBX* genes had at least one probe set. The average signal values of these genes from two or three biological replicates of each sample are shown in Table S3, and the hierarchical cluster analysis of their expression profile in ZS97 is illustrated in Figure 6A. Based on the hierarchical clustering, the expression profiles of the *OsBBX* genes can be divided into eight groups.

Group 1 consists of three genes (*OsBBX29*, *24* and *5*) that show relative high expressions in seedlings, young panicles and stamen. Group 2 contains four genes (*OsBBX27*, *11*, *4* and *2*) with relative high expression in seedlings, leaves and sheath, but with low transcript accumulation in stem and endosperms. *OsBBX21* and *15* in group 3 show relative high expression patterns in seedlings, panicles, stamen and endosperms. Nine genes (*OsBBX12*, *10*, *17*, *14*, *23*, *25*, *30*, *8* and *13*) belong to group 4. These genes have relative high expression level in most vegetative tissues as well as mature panicle and hull. Group 5 (*OsBBX9*, *3* and *20*) have relative high expression level in most tissues except developing young panicles and endosperms. Group 6 consists of three genes (*OsBBX26*, *6* and *22*) which have relative high expression level in seed, most of the vegetative tissues, mature panicle, hull or stamen. All the genes in group 7 (*OsBBX28*, *16* and *1*) have high expression level in seed, root and endosperms. The rest genes (*OsBBX19*, *7* and *18*) belong to group 8 and have relative high expression in vegetative tissues like leaves, hull and endosperms. To validate the results of the microarray expression analysis, a qPCR analysis was performed for several representative genes. The expression patterns of the selected genes were in general agreement with the microarray data (Figure 6B).

### Expression analysis of the *OsBBX* genes under light/dark treatment

**Figure 7:**
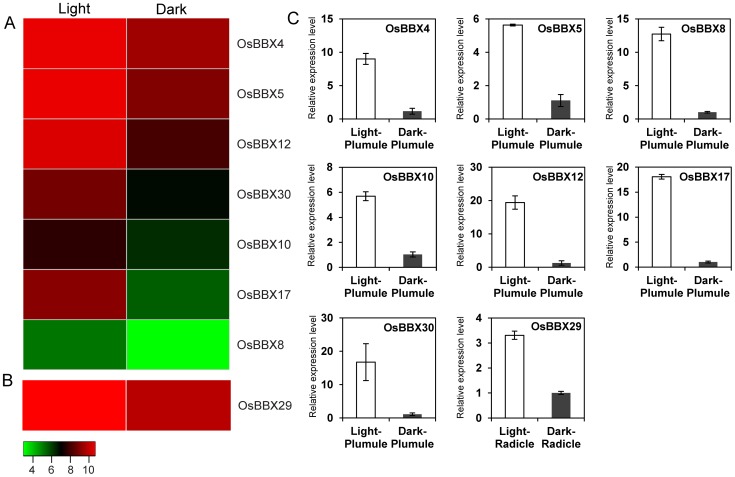
The differentially expressed *OsBBX* genes are regulated by light and dark treatments. (A) Differentially expressed genes in the plumule of 48 h after emergence with light/dark treatments. (B) Differentially expressed genes in the radicle of 48 h after emergence with light/dark treatments. The color key at the bottom represents the log_2_-based expression values. (C) The qPCR verification of all differentially expressed genes under the light/dark treatments. The x-axis indicates the treatments and tissues. The y-axis is the relative expression level of each gene. The error bars indicate the standard deviations of the independent biological replicates.

To investigate the possible light regulation of the *OsBBX* genes, the expression patterns of the *OsBBX* genes in the plumules and radicles under light/dark treatments were examined. Seven and one genes were differentially expressed in plumule and radicle, respectively (Figure 7A and 7B, Table S4). These eight genes (*OsBBX4*, *5*/*OsCOL4*, *8*, *10*, *12*, *17* and *30* in the plumule and *OsBBX29* in the radicle) were up-regulated under light compared to darkness. The most responsive gene to light is *OsBBX17*, which is up-regulated 12-fold when the plumule is exposed to light compared to its level under dark conditions. In the plumule, the *OsBBX8* and *OsBBX12* genes are up-regulated nearly 5-fold under light conditions compare to under darkness. All the differentially expressed genes under light/dark treatments were verified by qPCR and are listed in Figure 7C.

### Diurnal expression pattern analysis

**Figure 8:**
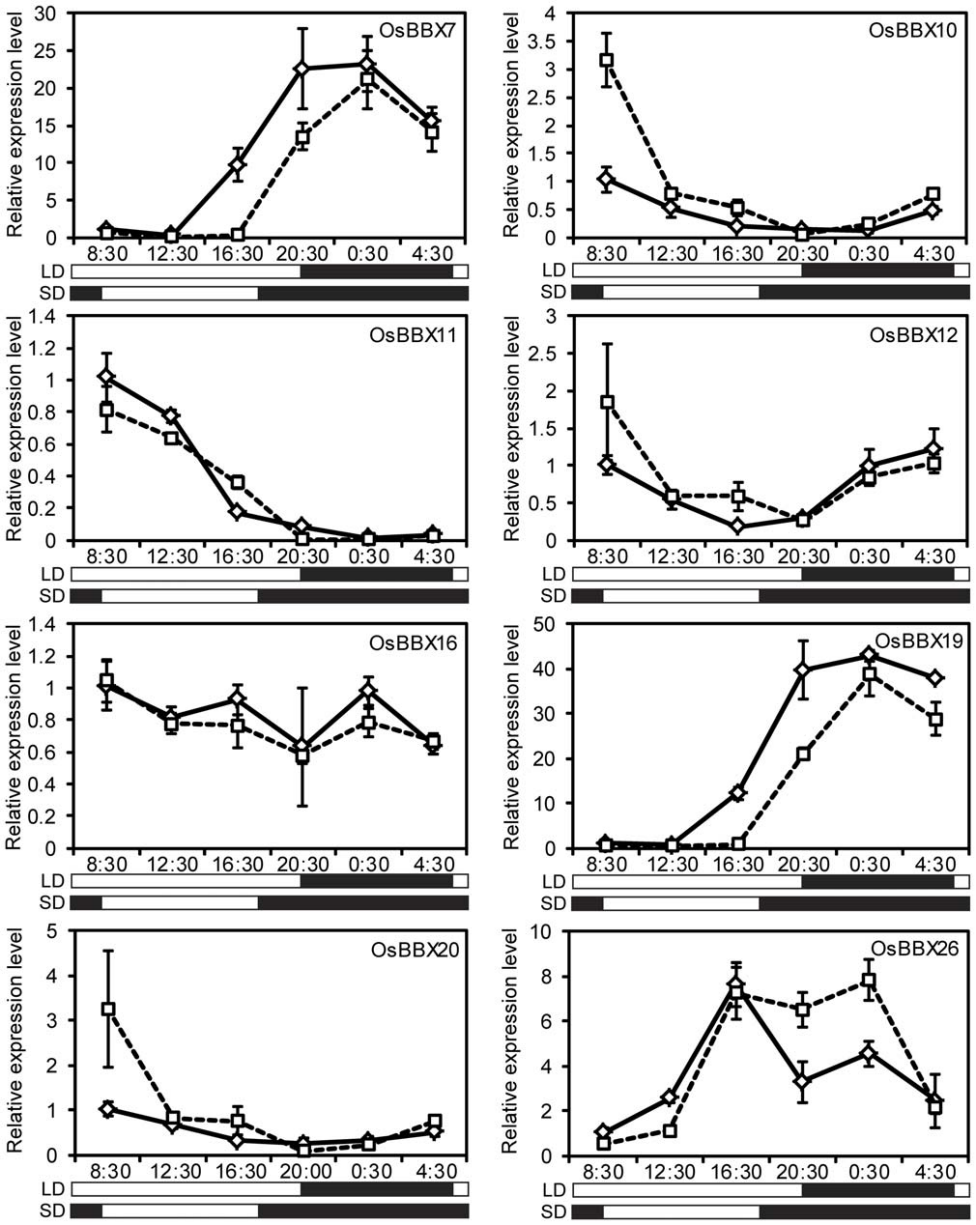
qPCR analysis of the diurnal expression patterns of the *OsBBX* genes. The white and black bars at the bottom indicate the light and dark periods, respectively. The numbers above the bars indicate the time of the day. The y-axis shows the relative transcript levels of each gene. The error bars indicate the standard deviations of the independent biological replicates.

Some *Arabidopsis BBX* genes have shown diurnal expression patterns. Therefore, we verified the diurnal expression patterns of the *OsBBX* genes using data from the Diurnal Project. The expression patterns of 29 *OsBBX* genes can be found in the Diurnal Project. Among these genes, 25 have a diurnal expression pattern (Figure S2). Eight *OsBBX* genes with relative high expression levels were selected for validation by qPCR. The qPCR results confirmed that seven of the eight genes had a diurnal expression pattern (Figure 8). *OsBBX7* and *OsBBX19* exhibited the same expression pattern which belonged to a pair of segmental duplication genes. Under SD conditions, the expression of both of these genes immediately increased at the beginning of the night, exhibited a peak at midnight, rapidly dropped towards the end of the night and had their lowest expression value at dawn. Under LD conditions, both gene transcripts were much more abundant during the light period than during the dark period. The expression of both the *OsBBX10* and *OsBBX20* genes was higher under SD than under LD conditions and peaked in the early morning under both photoperiodic conditions. The level of *OsBBX11* transcripts began to increase in the dark phase and peaked in the early morning under both photoperiodic conditions. The *OsBBX12* gene exhibited the same diurnal expression pattern in both photoperiodic conditions. The transcript of *OsBBX26* was much more abundant during the light period than during the dark period under LD conditions, whereas it was nearly the same under SD conditions. The *OsBBX16* gene did not show an obvious diurnal expression pattern.

### Response of the *OsBBX* genes to phytohormone treatments

**Figure 9:**
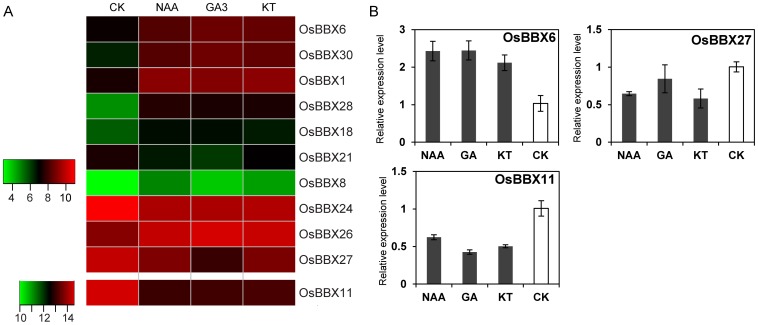
Expression profiles of the *OsBBX* genes in response to phytohormone treatments. (A) Hierarchical cluster displaying the differentially expressed *OsBBX* genes under NAA, GA_3_ and KT treatments. The color key at the left represents the log_2_-based expression values. (B) The qPCR verification of some of the differentially expressed *OsBBX* genes. The x-axis indicates the treatment. The y-axis shows the relative expression level of each treatment compared to control. The error bars indicate the standard deviations of the independent biological replicates.

To survey the *OsBBX* genes in response to phytohormone treatment, we also studied the responsiveness of the *OsBBX* genes to different phytohormone treatments, including NAA (a member of the auxin family), GA_3_ (a gibberellin) and KT (a cytokinin) using microarray analysis (Figure 9A). Eleven genes showed differential expression that was more than two-fold under at least one phytohormone treatment. Seven genes (*OsBBX1*, *6*, *11*, *24*, *26*, *27*/*OsCO3* and *30*) showed differential expression under the three phytohormone treatments, and three (*OsBBX11*, *24* and *27*) of these genes were down-regulated. One gene (*OsBBX8*) was up-regulated under NAA and KT treatments, one gene (*OsBBX21*) was down-regulated under GA_3_ treatment, and two genes (*OsBBX18* and *OsBBX28*) were up-regulated specifically in response to treatment with NAA. The fold-changes of the *OsBBX* genes that exhibited differential expression are shown in Table S4. We prepared new samples of rice plants that were exposed to phytohormone treatments and used these samples to verify the findings by qPCR; the results are shown in Figure 9B.

### In silico *cis*-element analysis of the *OsBBX* genes

A total of 90 putative *cis*-elements in the *OsBBX* upstream genomic sequences were identified by the PlantCARE database. Except the common *cis*-acting element in the promoter, enhancer regions (CAAT-box) and core promoter element around −30 of transcription start (TATA-box), 18 others *cis*-elements were found in more than 10 of the 30 *OsBBX*s which can be classified into four groups. Nine *cis*-elements that are involved in the light responsiveness of rice are: the circadian, G-box, Sp1, Box I, GT1-motif, Box 4, I-box, GAG-motif and TCCC-motif elements. Six of the *cis*-elements are well-characterized stress-responsive elements: HSE, ARE, W box, GC-motif, Box-W1 and MBS elements. The three *cis*-acting regulatory elements that are involved in hormone responsiveness are: ABRE, TGACG-motif and CGTCA-motif elements. One *cis*-acting regulatory element (Skn-1_motif) is required for endosperm expression. The *cis*-elements that were identified among the 30 *OsBBX* genes and the putative functions and positions of these elements are listed in Table 2 and Table S5, respectively.

**Table 2:** The *cis*-elements that have been identified in more than ten *OsBBX* genes.

*Cis*-elements	Number of genes	Functions of the *cis*-elements	*Cis*-elements types
CAAT-box	30	Common *cis*-acting element in promoter and enhancer regions	
TATA-box	30	Core promoter element around −30 of transcription start	
Sp1	28	Light responsive element	Light responsive
G-box	27	*Cis*-acting regulatory element involved in light responsiveness	Light responsive
ABRE	21	*Cis*-acting element involved in the abs*cis*ic acid responsiveness	Hormone responsive
TGACG-motif	20	*Cis*-acting regulatory element involved in the meja-responsiveness	Hormone responsive
ARE	19	*Cis*-acting regulatory element essential for the anaerobic induction	Stress responsive
CGTCA-motif	19	*Cis*-acting regulatory element involved in the meja-responsiveness	Hormone responsive
Skn-1_motif	19	*Cis*-acting regulatory element required for endosperm expression	Endosperm expression
I-box	18	Part of a light responsive element	Light responsive
circadian	16	*Cis*-acting regulatory element involved in circadian control	Light responsive
W box	14	Wounding and pathogen responsievness.	Stress responsive
GC-motif	14	Enhancer-like element involved in anoxic specific inducibility	Stress responsive
Box-W1	14	Fungal elicitor responsive element	Stress responsive
Box 4	14	Part of a conserved DNA module involved in light responsiveness	Light responsive
GAG-motif	13	Part of a light responsive element	Light responsive
HSE	12	*Cis*-acting element involved in heat stress responsiveness	Stress responsive
Box I	12	Light responsive element	Light responsive
MBS	12	MYB binding site involved in drought-inducibility	Stress responsive
GT1-motif	11	Light responsive element	Light responsive
TCCC-motif	11	Part of a light responsive element	Light responsive

## Discussion

### Evolution of the *OsBBX* gene family

The rice genome size and gene number are approximately 3.7 times (450 Mb vs. 130 Mb) and 1.5 times (37,000 vs. 25,000) larger, respectively, than those of *Arabidopsis* [50]. However, we identified fewer *BBX* genes in rice than in *Arabidopsis*. The reason for this discrepancy could be the variable status of genome duplications in *Arabidopsis* and rice [51], [52]. Eighteen *OsBBX* genes are involved in segmental duplication events of the rice chromosome, fourteen of which contains one CCT domain and either one or two B-box domains, two with two B-box domains, and two genes with only one B-box domain. The rice *BBX* gene family contains 17 OsBBXs with one or two B-box domains and one CCT domain, which is similar to the genes found in *Arabidopsis*. Ten OsBBXs contained two B-box domains, and three OsBBXs contain only one B-box domain; in contrast, eight AtBBXs contain two B-box domains, and seven AtBBX*s* contain only one B-box domain. These results suggest that the rice and *Arabidopsis BBX* genes probably had a common ancestor and that the expansion occurred independently after the divergence of the monocots and the dicots.

During the course of plant evolution, tandem duplication and large-scale segmental duplication play an important role in maintaining the large number of genes that belong to specific gene families [53]. To elucidate the potential mechanism of evolution of the *OsBBX* gene family, both the tandem and segmental duplication events were analyzed. Although no tandem duplication was found, 18 *OsBBXs* participated in segmental duplication. These results suggest that segmental duplication events contributed significantly to the expansion of the *OsBBX* gene family. The same potential mechanism of gene family evolution was also found in the *bZIP* transcription factor gene family in rice [54].

A phylogenic tree of the BBXs from rice, *Arabidopsis*, maize, poplar and *sorghum bicolor* was constructed. In the above mentioned tree, the phylogenic relationships among the OsBBXs were almost the same as in a phylogenic tree that contained only the BBXs from rice. Moreover, many of the BBXs from the monocot were clustered together, which indicated that these genes were orthologous; this finding is consistent with the results reported by Griffiths et al [24]. The phylogenic tree that contains the OsBBX and AtBBX showed that most of the BBXs are clustered in species-specific clades, indicating that the BBXs evolved separately after the monocot and dicot divergence (Figure S3). Griffiths et al [24] suggested that the monocot plants have a unique subfamily that contains one B-box motif and a CCT motif that is derived through the internal deletion of the second B-box domain. The position of OsBBX25 and OsBBX27 in the phylogenic trees confirmed this conclusion. The sequence alignment showed that the OsBBX7 and OsBBX19 possessed the second B-box domains, which indicated that these proteins diverged from the other BBXs that contain two B-box domains. The aligned B-box 1, B-Box 2, CCT and concatenated B-Box and CCT domain sequences were also used for phylogenetic analysis. Similar overall patterns compared with the full-length sequence tree were observed. These results indicate that the B-Box and CCT domains may be relevant for the conserved functions of the BBX family.

### Divergent expression profile of the *OsBBX* genes

Microarray analysis is a high-throughput method that can be used to analyze the expression pattern of many genes at the transcription level. Our analyses indicated that the expression patterns of the 30 *OsBBX* genes could be classified into eight groups. From the phylogenetic analysis, we identified several paralogous *OsBBXs*. Some paralogous genes (*OsBBX3*/*OsBBX20*, *OsBBX4*/*OsBBX11*, *OsBBX7*/*OsBBX19* and *OsBBX8*/*OsBBX10*/*OsBBX17*) have similar expression patterns. Therefore, we inferred that these genes had obtained redundant expression pattern during the evolution. On the other hand, some paralogous genes (*OsBBX1*/*OsBBX14*, *OsBBX2*/*OsBBX21*, *OsBBX5*/*OsBBX12*, *OsBBX6*/*OsBBX13*, *OsBBX9*/*OsBBX23* and *OsBBX26*/*OsBBX28*) have divergent expression patterns indicating that these genes had evolutionarily obtained unique expression pattern.

The development of comparative genomics has made possible to analyze the genes of the same gene family among different species. The phylogenetic analysis indicated that most OsBBXs and AtBBXs are clustered in species-specific clades with high bootstrap supporting, except for four sets of orthologous genes: *OsBBX16* and *AtBBX22*; *OsBBX29*, *AtBBX18* and *AtBBX19*; *OsBBX24*, *AtBBX28* and *AtBBX29*; *OsBBX18*, *AtBBX1*, *AtBBX2* and *AtBBX3*. The *AtBBX22*, *AtBBX18* (*DBB1a*) and At*BBX19* (*DBB1b*) are positive or negative regulate genes of light signaling in *Arabidopsis* [9], [10], [19]. Therefore, we inferred that *OsBBX16* and *OsBBX29* might participate in the light signaling pathway even though *OsBBX16* did not have an obviously diurnal expression pattern. Previous studies showed that the expression of *OsBBX29* oscillates in continuous light [10].

### Light regulation of the *OsBBX* genes and *cis*-elements analysis

Light is one of the major environmental factors that regulate plant growth and development. Plants perceive various light signals and subsequently regulate diverse developmental processes, such as skotomorphogenesis, photomorphogenesis, shade avoidance, circadian growth and flowering time control. Skotomorphogenesis and photomorphogenesis are two developmental processes that occur in plants that grow in the dark and light, respectively. The proper regulation of these two stages is important for the optimization of plant growth and for their success in response to the environment [55]. Seedlings at the plumule and radicle stages were placed under 48 h of continuous light or darkness, and samples were collected for microarray experiments. Analysis of the microarray data showed that eight genes were differentially regulated by the light and dark treatments. All eight genes were up-regulated under light conditions compared to darkness. In addition, *OsBBX5* (*OsCOL4*) also exhibited differential expression in our microarray analysis. Therefore, we inferred that these differentially expressed genes might play a role in the light signaling pathway, especially in the early photomorphogenesis stage in rice. Previous reports have suggested that many *BBX* genes are implicated in a light signal transduction pathway [9], [10], [15], [16], [18], [19]. The analysis of data obtained from the Diurnal Project and the qPCR data showed that most of the rice *BBX* genes had diurnal expression patterns, suggesting that the *OsBBX* family members may be involved in the light signaling pathway in rice.

A total of 90 *cis*-acting regulatory elements were identified. The frequency of light-responsive *cis*-regulatory elements in the *OsBBX* promoter region ranges from three to seven. *OsBBX6* had the maximum number of light-responsive *cis*-elements followed by *OsBBX1*, *5*, *8*, *10*, *16*, *25*, *26* and *27*, which have six light-responsive *cis*-elements. The light-responsive element Sp1 was found in the promoter region of 28 *OsBBX* genes. The *cis*-element circadian was found in the promoter region of 16 *OsBBX*s, including *OsBBX7*, *10*, *11*, *19*, *26* and *27*. All of these genes exhibit the diurnal expression patterns. Therefore, we inferred that the genes with the circadian *cis*-element may have a diurnal expression pattern that is regulated by light. The presence of many light responsive elements in the promoter region of the *OsBBX* genes strongly suggests the involvement of these *BBX* genes in the regulation of the photoperiodic control of flowering.

The majority of the *OsBBX* genes have hormone-responsive *cis*-elements, except *OsBBX*1, *OsBBX*14 and *OsBBX*19, which indicates that these genes have a putative role in hormone regulation. Twenty-nine *OsBBX* genes contain at least one of the stress-responsive *cis*-elements (ARE, W box, GC-motif, Box-W1, HSE and MBS), suggesting that these genes have a putative role in the response to abiotic and biotic stress. The identified *cis*-elements are mainly related to five important physiological phenomena: light, hormone and stress responsiveness and endosperm- and meristem-specific gene expression. Their presence in the upstream sequence of *OsBBX* genes indicates that the OsBBX members have additional unknown functions.

### Phytohormonal regulation of *OsBBX* genes

Plant hormones are essential for plant growth and development. Auxin, GA, cytokinin, ABA and ethylene are the ‘classical’ phytohormones. Notably, these hormones can regulate many processes independently. However, their overlapping influence on various cellular processes suggests that there is cooperation and crosstalk between their signaling pathways [56]. Furthermore, an increasing number of studies showed that a tight crosstalk between light and hormones to regulates seed germination and seedling photomorphogenesis, and can lead to a dramatic change in plant morphology [57]. Previous studies have shown that the *BBX* genes participate in light signaling and hormone responses. *DBB1a* functions as a negative regulator of blue light-mediated hypocotyl elongation and is involved in gibberellin homeostasis in *Arabidopsis* [15]. *AtBBX22* may play a role in regulating light signaling and hormone responses [20]. Our results showed that eleven *OsBBX* genes are regulated by hormones. Eight of these genes are regulated by more than one hormone treatment, which suggests that these genes may be involved in the interaction of different hormone signals at the physiological level. In addition, three genes (*OsBBX8*, *27* and *30*) responded to both hormone and light treatments, which indicated that these genes may integrate light and hormone signals. Further characterization of additional *OsBBX* genes that are involved in different hormone and light responses will greatly expand our understanding of the functions of the *OsBBX* genes and the crosstalk that occurs between the hormone and light signaling pathways.

In conclusion, this study provided a genomic framework, uniform nomenclature and phylogenetic analysis of the 30 *OsBBX* genes. In addition, we studied the expression profile of these 30 *OsBBX* genes during the whole life-cycle of rice and under phytohormone and light/dark treatments. Furthermore, the diurnal expression patterns of the *OsBBX* genes were also investigated. Our investigation provides insight into the role of *OsBBX* genes in certain developmental stages and in the phytohormone and light signaling pathways. Our analysis provides a useful reference for more detailed functional analyses of these *BBX* genes in rice and will be helpful in the selection of appropriate candidate genes for further study.

## Supporting Information

Figure S1 **Gene structure of each** ***OsBBX*** **gene.** The white rectangles represent the exons and the black lines represent the introns. The UTR regions are marked as black rectangles.
(TIF)

Figure S2 **Diurnal expression pattern of** ***OsBBX*** **genes from the Diurnal Project.** The x-axis represents the time course over two days. The y-axis represents the average expression values obtained from the microarrays. The blue line represents the LDHC condition and the green line represents the LLHH (LDHC) condition.
(TIF)

Figure S3 **Phylogenetic analysis of the OsBBX and AtBBX members.** The unrooted tree was generated from the OsBBX and AtBBX full length protein sequences. The bootstrap values from 1000 replicates are indicated at each node. The triangles in front of the members indicate the predicted paralogous proteins. The members marked in red contain two B-box domains and one CCT domains. The scale bar represents 0.1 amino acid substitutions per site.
(PDF)

Figure S4 **Phylogenetic analysis of the B-box TFs from rice,** ***Arabidopsis*** **, maize, poplar and** ***sorghum bicolor*** **.** The sequences of the maize (Zm: *Zea Mays*), poplar ( Pt: *Populus trichocarpa*) and sorghum bicolor (Sb: *Sorghum bicolor*) BBX proteins were identified and downloaded from NCBI. The unrooted tree was generated from the full length protein sequences. The bootstrap values from 1000 replicates are indicated at each node. The scale bar represents 0.1 amino acid substitutions per site.
(PDF)

Table S1 **Detailed information of the rice samples used in the microarray analysis.**
(DOC)

Table S2 **Primers used for the qPCR analysis.**
(DOC)

Table S3 **Average expression signal values of the 30** ***OsBBX*** **genes.**
(XLS)

Table S4 **Results of the differential expression analysis of the** ***OsBBX*** **genes in the plumule and radicle under NAA, GA_3_ and KT treatment and light/dark regulation.**
(XLS)

Table S5 **The positions of the** ***cis*** **-elements that were identified in more than ten** ***OsBBX*** **genes.**
(XLS)

## References

[1] Gong W, Shen YP, Ma LG, Pan Y, Du YL, et al (2004) Genome-wide ORFeome cloning and analysis of *Arabidopsis* transcription factor genes. Plant Physiol 135: 773–782. 15208423 10.1104/pp.104.042176 PMC514114

[2] Gao G, Zhong YF, Guo AY, Zhu QH, Tang W, et al (2006) DRTF: a database of rice transcription factors. Bioinformatics 22: 1286–1287. 16551659 10.1093/bioinformatics/btl107

[3] Khanna R, Kronmiller B, Maszle DR, Coupland G, Holm M, et al (2009) The *Arabidopsis* B-box zinc finger family. Plant Cell 21: 3416–3420. 19920209 10.1105/tpc.109.069088 PMC2798317

[4] Klug A, Schwabe JW (1995) Protein motifs 5. Zinc fingers. FASEB J 9: 597–604. 7768350

[5] Putterill J, Robson F, Lee K, Simon R, Coupland G (1995) The *CONSTANS* gene of *Arabidopsis* promotes flowering and encodes a protein showing similarities to zinc finger transcription factors. Cell 80: 847–857. 7697715 10.1016/0092-8674(95)90288-0

[6] Robson F, Costa MM, Hepworth SR, Vizir I, Pineiro M, et al (2001) Functional importance of conserved domains in the flowering-time gene *CONSTANS* demonstrated by analysis of mutant alleles and transgenic plants. Plant J 28: 619–631. 11851908 10.1046/j.1365-313x.2001.01163.x

[7] Borden KL (1998) RING fingers and B-boxes: zinc-binding protein-protein interaction domains. Biochem Cell Biol 76: 351–358. 9923704 10.1139/bcb-76-2-3-351

[8] Torok M, Etkin LD (2000) Two B or not two B? Overview of the rapidly expanding B-box family of proteins. Differentiation 67: 63–71. 10.1046/j.1432-0436.2001.067003063.x 11428128

[9] Chang CS, Li YH, Chen LT, Chen WC, Hsieh WP, et al (2008) LZF1, a HY5-regulated transcriptional factor, functions in *Arabidopsis* de-etiolation. Plant J 54: 205–219. 18182030 10.1111/j.1365-313X.2008.03401.x

[10] Kumagai T, Ito S, Nakamichi N, Niwa Y, Murakami M, et al (2008) The common function of a novel subfamily of B-Box zinc finger proteins with reference to circadian-associated events in *Arabidopsis thaliana* . Biosci Biotechnol Biochem 72: 1539–1549. 18540109 10.1271/bbb.80041

[11] Ledger S, Strayer C, Ashton F, Kay SA, Putterill J (2001) Analysis of the function of two circadian-regulated *CONSTANS-LIKE* genes. Plant J 26: 15–22. 11359606 10.1046/j.1365-313x.2001.01003.x

[12] Datta S, Hettiarachchi GH, Deng XW, Holm M (2006) *Arabidopsis CONSTANS-LIKE3* is a positive regulator of red light signaling and root growth. Plant Cell 18: 70–84. 16339850 10.1105/tpc.105.038182 PMC1323485

[13] Hassidim M, Harir Y, Yakir E, Kron I, Green RM (2009) Over-expression of *CONSTANS-LIKE 5* can induce flowering in short-day grown *Arabidopsis* . Planta 230: 481–491. 19504268 10.1007/s00425-009-0958-7

[14] Cheng X-F, Wang Z-Y (2005) Overexpression of *COL9*, a *CONSTANS-LIKE* gene, delays flowering by reducing expression of *CO* and *FT* in *Arabidopsis thaliana* . Plant J 43: 758–768. 16115071 10.1111/j.1365-313X.2005.02491.x

[15] Wang QM, Zeng JX, Deng KQ, Tu XJ, Zhao XY, et al (2011) *DBB1a*, involved in gibberellin homeostasis, functions as a negative regulator of blue light-mediated hypocotyl elongation in *Arabidopsis* . Planta 233: 13–23. 20872270 10.1007/s00425-010-1274-y

[16] Indorf M, Cordero J, Neuhaus G, Rodríguez-Franco M (2007) Salt tolerance (STO), a stress-related protein, has a major role in light signalling. Plant J 51: 563–574. 17605755 10.1111/j.1365-313X.2007.03162.x

[17] Holm M, Hardtke CS, Gaudet R, Deng X-W (2001) Identification of a structural motif that confers specific interaction with the WD40 repeat domain of *Arabidopsis* COP1. EMBO J 20: 118–127. 11226162 10.1093/emboj/20.1.118 PMC140188

[18] Datta S, Hettiarachchi C, Johansson H, Holm M (2007) SALT TOLERANCE HOMOLOG2, a B-box protein in *Arabidopsis* that activates transcription and positively regulates light-mediated development. Plant Cell 19: 3242–3255. 17965270 10.1105/tpc.107.054791 PMC2174709

[19] Datta S, Johansson H, Hettiarachchi C, Irigoyen ML, Desai M, et al (2008) LZF1/SALT TOLERANCE HOMOLOG3, an *Arabidopsis* B-box protein involved in light-dependent development and gene expression, undergoes COP1-mediated ubiquitination. Plant Cell 20: 2324–2338. 18796637 10.1105/tpc.108.061747 PMC2570732

[20] Chang C-SJ, Maloof JN, Wu S-H (2011) COP1-mediated degradation of BBX22/LZF1 optimizes seedling development in *Arabidopsis* . Plant Physiol 156: 228–239. 21427283 10.1104/pp.111.175042 PMC3091042

[21] Holtan HE, Bandong S, Marion CM, Adam L, Tiwari S, et al (2011) BBX32, an *Arabidopsis* b-box protein, functions in light signaling by suppressing hy5-regulated gene expression and interacting with STH2/BBX21. Plant Physiol 156: 2109–2123. 21632973 10.1104/pp.111.177139 PMC3149924

[22] Yano M, Katayose Y, Ashikari M, Yamanouchi U, Monna L, et al (2000) *Hd1*, a major photoperiod sensitivity quantitative trait locus in rice, is closely related to the Arabidopsis flowering time gene *CONSTANS* . Plant Cell 12: 2473–2484. 11148291 10.1105/tpc.12.12.2473 PMC102231

[23] Kim SK, Yun CH, Lee JH, Jang YH, Park HY, et al (2008) *OsCO3*, a *CONSTANS-LIKE* gene, controls flowering by negatively regulating the expression of *FT*-like genes under SD conditions in rice. Planta 228: 355–365. 18449564 10.1007/s00425-008-0742-0

[24] Griffiths S, Dunford RP, Coupland G, Laurie DA (2003) The evolution of *CONSTANS*-like gene families in barley, rice, and Arabidopsis. Plant Physiol 131: 1855–1867. 12692345 10.1104/pp.102.016188 PMC166942

[25] Lee YS, Jeong DH, Lee DY, Yi J, Ryu CH, et al (2010) *OsCOL4* is a constitutive flowering repressor upstream of *Ehd1* and downstream of *OsphyB* . Plant J 63: 18–30. 20409004 10.1111/j.1365-313X.2010.04226.x

[26] Altschul SF, Madden TL, Schäffer AA, Zhang J, Zhang Z, et al (1997) Gapped BLAST and PSI-BLAST: a new generation of protein database search programs. Nucleic Acids Res 25: 3389–3402. 9254694 10.1093/nar/25.17.3389 PMC146917

[27] Ouyang S, Zhu W, Hamilton J, Lin H, Campbell M, et al (2007) The TIGR Rice Genome Annotation Resource: improvements and new features. Nucleic Acids Res 35: D883–887. 17145706 10.1093/nar/gkl976 PMC1751532

[28] Tanaka T, Antonio BA, Kikuchi S, Matsumoto T, Nagamura Y, et al (2008) The Rice Annotation Project Database (RAP-DB): 2008 update. Nucleic Acids Res 36: D1028–D1033. 18089549 10.1093/nar/gkm978 PMC2238920

[29] Itoh T, Tanaka T, Barrero RA, Yamasaki C, Fujii Y, et al (2007) Curated genome annotation of *Oryza sativa* ssp. *japonica* and comparative genome analysis with *Arabidopsis thaliana* . Genome Res 17: 175–183. 17210932 10.1101/gr.5509507 PMC1781349

[30] Kikuchi S, Satoh K, Nagata T, Kawagashira N, Doi K, et al (2003) Collection, mapping, and annotation of over 28,000 cDNA clones from *japonica* rice. Science 301: 376–379. 12869764 10.1126/science.1081288

[31] Schultz J, Milpetz F, Bork P, Ponting CP (1998) SMART, a simple modular architecture research tool: identification of signaling domains. Proc Natl Acad Sci U S A 95: 5857–5864. 9600884 10.1073/pnas.95.11.5857 PMC34487

[32] Letunic I, Doerks T, Bork P (2012) SMART 7: recent updates to the protein domain annotation resource. Nucleic Acids Res 40: D302–305. 22053084 10.1093/nar/gkr931 PMC3245027

[33] Punta M, Coggill PC, Eberhardt RY, Mistry J, Tate J, et al (2012) The Pfam protein families database. Nucleic Acids Res 40: D290–D301. 22127870 10.1093/nar/gkr1065 PMC3245129

[34] Guo AY, Zhu QH, Chen X, Luo JC (2007) GSDS: a gene structure display server. Yi chuan 29: 1023–1026. 17681935

[35] Voorrips RE (2002) MapChart: software for the graphical presentation of linkage maps and QTLs. J Hered 93: 77–78. 12011185 10.1093/jhered/93.1.77

[36] Tamura K, Peterson D, Peterson N, Stecher G, Nei M, et al (2011) MEGA5: molecular evolutionary genetics analysis using maximum likelihood, evolutionary distance, and maximum parsimony methods. Mol Biol Evol 28: 2731–2739. 21546353 10.1093/molbev/msr121 PMC3203626

[37] Crooks GE, Hon G, Chandonia JM, Brenner SE (2004) WebLogo: a sequence logo generator. Genome Res 14: 1188–1190. 15173120 10.1101/gr.849004 PMC419797

[38] Wang L, Xie W, Chen Y, Tang W, Yang J, et al (2010) A dynamic gene expression atlas covering the entire life cycle of rice. Plant J 61: 752–766. 20003165 10.1111/j.1365-313X.2009.04100.x

[39] Zhao X, Huang J, Yu H, Wang L, Xie W (2010) Genomic survey, characterization and expression profile analysis of the peptide transporter family in rice (*Oryza sativa* L.). BMC Plant Biol 10: 92. 20487558 10.1186/1471-2229-10-92 PMC3017762

[40] Livak KJ, Schmittgen TD (2001) Analysis of relative gene expression data using real-time quantitative PCR and the 2(−Delta Delta C(T)) Method. Methods 25: 402–408. 11846609 10.1006/meth.2001.1262

[41] Mockler TC, Michael TP, Priest HD, Shen R, Sullivan CM, et al (2007) The DIURNAL project: DIURNAL and circadian expression profiling, model-based pattern matching, and promoter analysis. Cold Spring Harb Symp Quant Biol 72: 353–363. 18419293 10.1101/sqb.2007.72.006

[42] Michael TP, Mockler TC, Breton G, McEntee C, Byer A, et al (2008) Network discovery pipeline elucidates conserved time-of-day-specific cis-regulatory modules. PLoS Genet 4: e14. 18248097 10.1371/journal.pgen.0040014 PMC2222925

[43] Xue W, Xing Y, Weng X, Zhao Y, Tang W, et al (2008) Natural variation in *Ghd7* is an important regulator of heading date and yield potential in rice. Nat Genet 40: 761–767. 18454147 10.1038/ng.143

[44] Lescot M, Dehais P, Thijs G, Marchal K, Moreau Y, et al (2002) PlantCARE, a database of plant *cis*-acting regulatory elements and a portal to tools for *in silico* analysis of promoter sequences. Nucleic Acids Res 30: 325–327. 11752327 10.1093/nar/30.1.325 PMC99092

[45] Zhang H, Jin J, Tang L, Zhao Y, Gu X, et al (2011) PlantTFDB 2.0: update and improvement of the comprehensive plant transcription factor database. Nucleic Acids Res 39: D1114–1117. 21097470 10.1093/nar/gkq1141 PMC3013715

[46] Perez-Rodriguez P, Riano-Pachon DM, Correa LGG, Rensing SA, Kersten B, et al (2010) PInTFDB: updated content and new features of the plant transcription factor database. Nucleic Acids Res 38: D822–D827. 19858103 10.1093/nar/gkp805 PMC2808933

[47] Mochida K, Yoshida T, Sakurai T, Yamaguchi-Shinozaki K, Shinozaki K, et al (2011) *In silico* analysis of transcription factor repertoires and prediction of stress-responsive transcription factors from six major gramineae plants. DNA Res 18: 321–332. 21729923 10.1093/dnares/dsr019 PMC3190953

[48] Yilmaz A, Nishiyama MY Jr, Fuentes BG, Souza GM, Janies D, et al (2009) GRASSIUS: a platform for comparative regulatory genomics across the grasses. Plant Physiol 149: 171–180. 18987217 10.1104/pp.108.128579 PMC2613736

[49] Gray J, Bevan M, Brutnell T, Buell CR, Cone K, et al (2009) A recommendation for naming transcription factor proteins in the grasses. Plant Physiol 149: 4–6. 19126689 10.1104/pp.108.128504 PMC2613739

[50] Vij S, Gupta V, Kumar D, Vydianathan R, Raghuvanshi S, et al (2006) Decoding the rice genome. Bioessays 28: 421–432. 16547947 10.1002/bies.20399

[51] Paterson AH, Bowers JE, Chapman BA (2004) Ancient polyploidization predating divergence of the cereals, and its consequences for comparative genomics. Proc Natl Acad Sci U S A 101: 9903–9908. 15161969 10.1073/pnas.0307901101 PMC470771

[52] Yu J, Wang J, Lin W, Li S, Li H, et al (2005) The Genomes of *Oryza sativa*: a history of duplications. PLoS Biol 3: e38. 15685292 10.1371/journal.pbio.0030038 PMC546038

[53] Cannon SB, Mitra A, Baumgarten A, Young ND, May G (2004) The roles of segmental and tandem gene duplication in the evolution of large gene families in Arabidopsis thaliana. BMC Plant Biol 4: 10. 15171794 10.1186/1471-2229-4-10 PMC446195

[54] Nijhawan A, Jain M, Tyagi AK, Khurana JP (2008) Genomic survey and gene expression analysis of the basic leucine zipper transcription factor family in rice. Plant Physiol 146: 333–350. 18065552 10.1104/pp.107.112821 PMC2245831

[55] Casal JJ, Fankhauser C, Coupland G, Blázquez MA (2004) Signalling for developmental plasticity. Trends Plant Sci 9: 309–314. 15165563 10.1016/j.tplants.2004.04.007

[56] Santner A, Calderon-Villalobos LIA, Estelle M (2009) Plant hormones are versatile chemical regulators of plant growth. Nat Chem Biol 5: 301–307. 19377456 10.1038/nchembio.165

[57] Lau OS, Deng XW (2010) Plant hormone signaling lightens up: integrators of light and hormones. Curr Opin Plant Biol 13: 571–577. 20739215 10.1016/j.pbi.2010.07.001

