# Supplementary figures and images for: The Rice B-Box Zinc Finger Gene Family: Genomic Identification, Characterization, Expression Profiling and Diurnal Analysis
Source: PLoS One. 2012 Oct 31;7(10):e48242. doi: 10.1371/journal.pone.0048242 (PMC3485221; doi:10.1371/journal.pone.0048242)

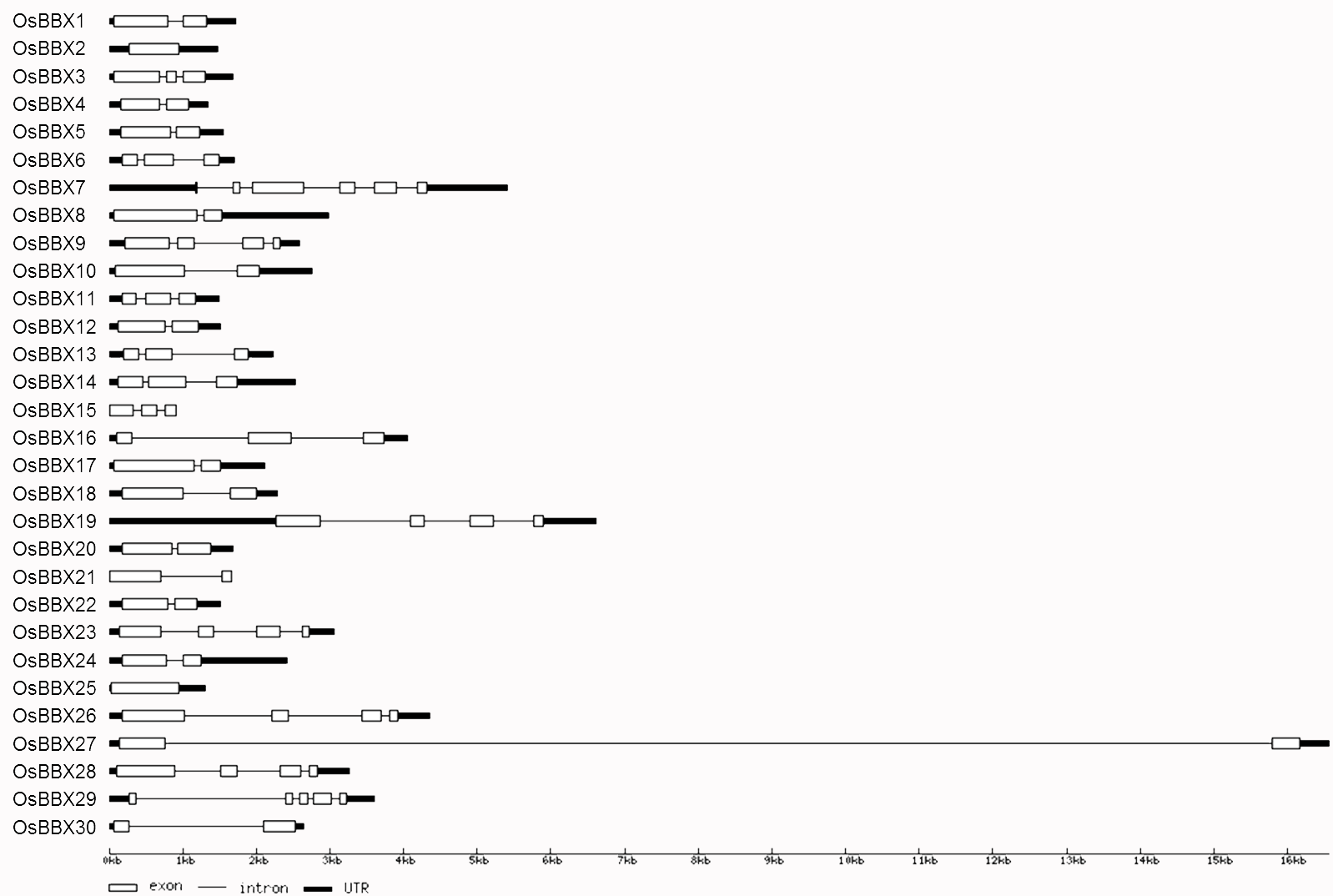

Supplement: Figure S1 — Gene structure of each OsBBX gene. The white rectangles represent the exons and the black lines represent the introns. The UTR regions are marked as black rectangles. (TIF) [file pone.0048242.s001.tif]

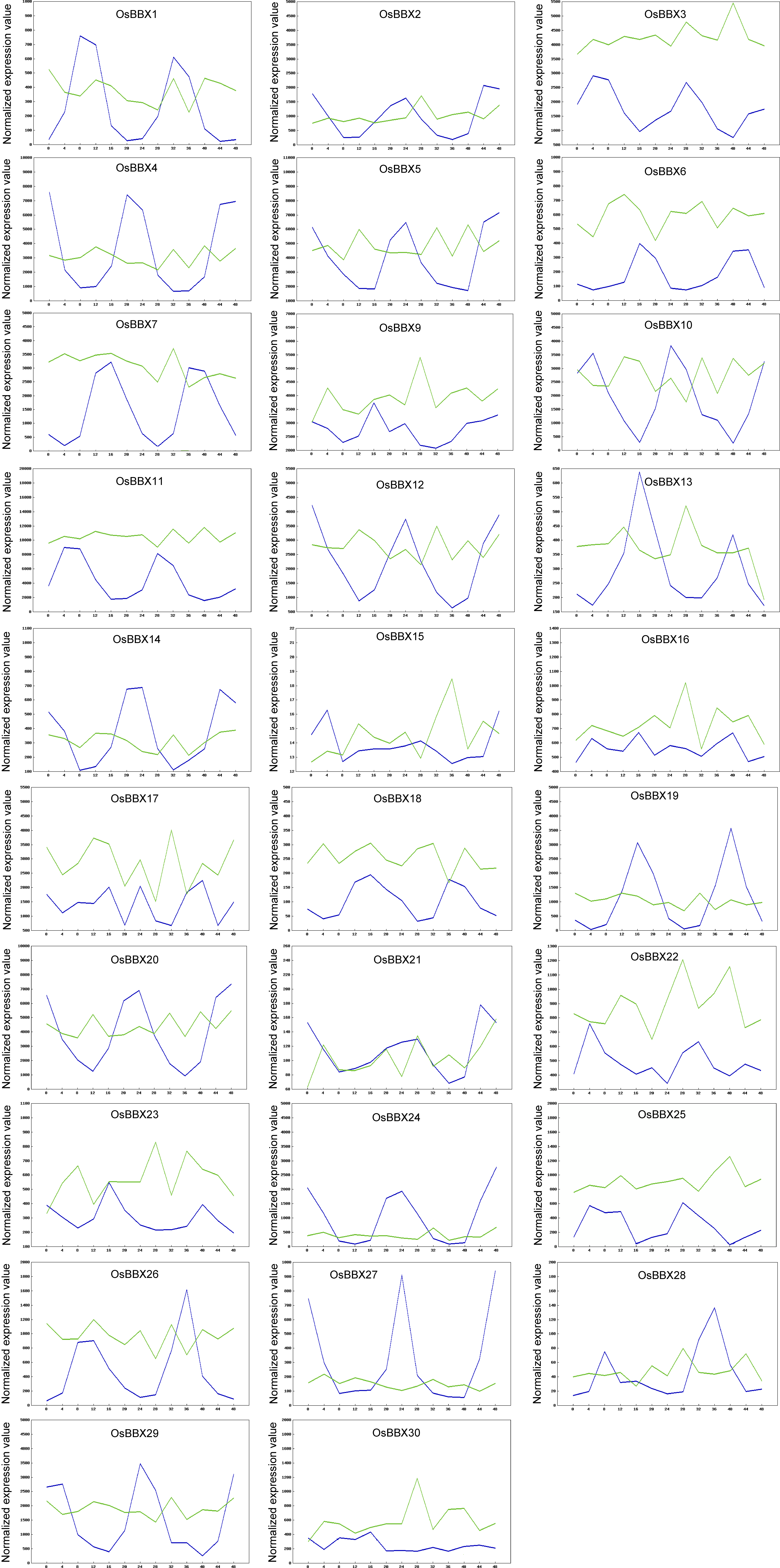

Supplement: Figure S2 — Diurnal expression pattern of OsBBX genes from the Diurnal Project. The x-axis represents the time course over two days. The y-axis represents the average expression values obtained from the microarrays. The blue line represents the LDHC condition and the green line represents the LLHH (LDHC) condition. (TIF) [file pone.0048242.s002.tif]

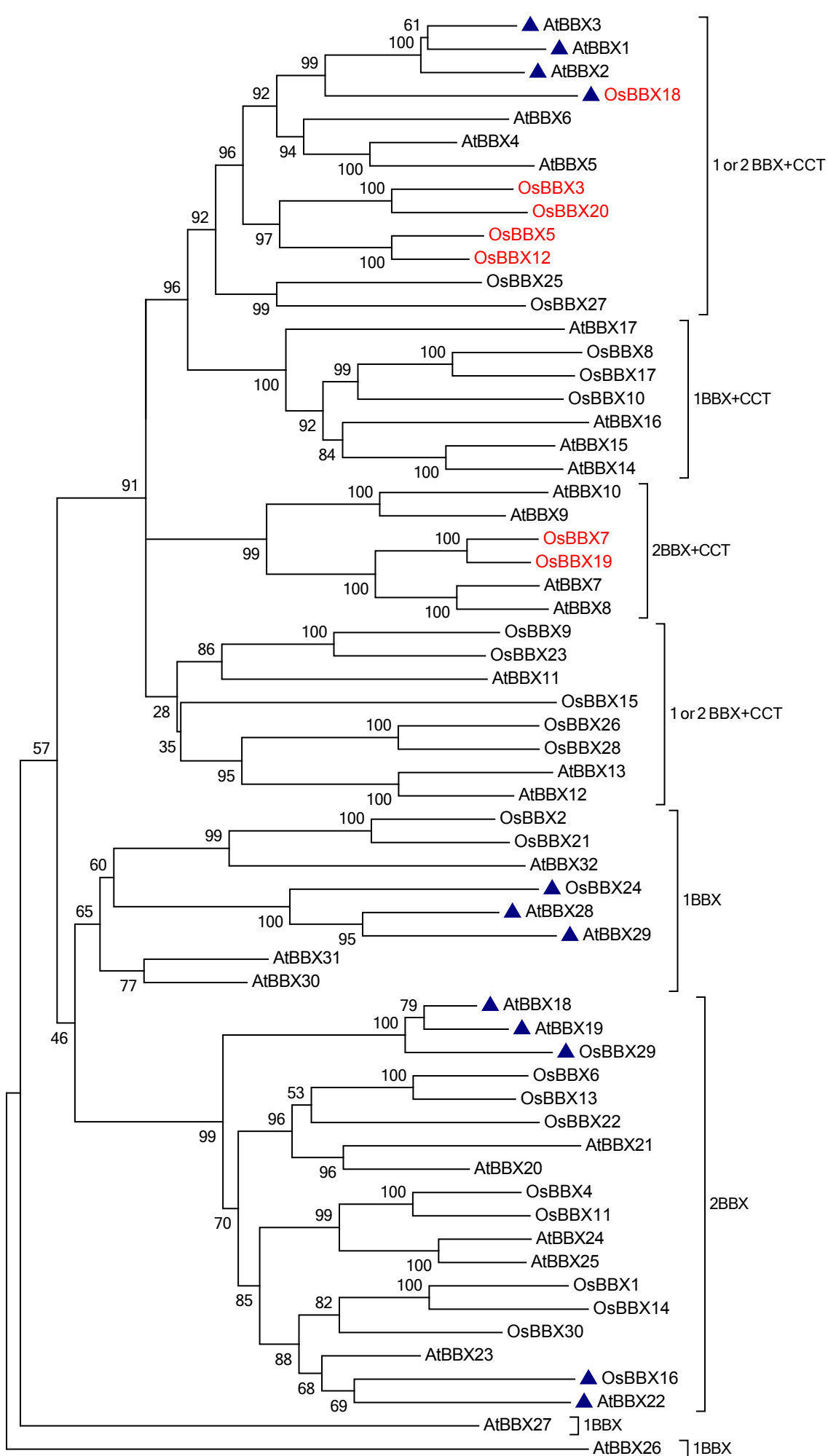

Supplement: Figure S3 — Phylogenetic analysis of the OsBBX and AtBBX members. The unrooted tree was generated from the OsBBX and AtBBX full length protein sequences. The bootstrap values from 1000 replicates are indicated at each node. The triangles in front of the members indicate the predicted paralogous proteins. The members marked in red contain two B-box domains and one CCT domains. The scale bar represents 0.1 amino acid substitutions per site. (PDF) [file pone.0048242.s003.pdf]

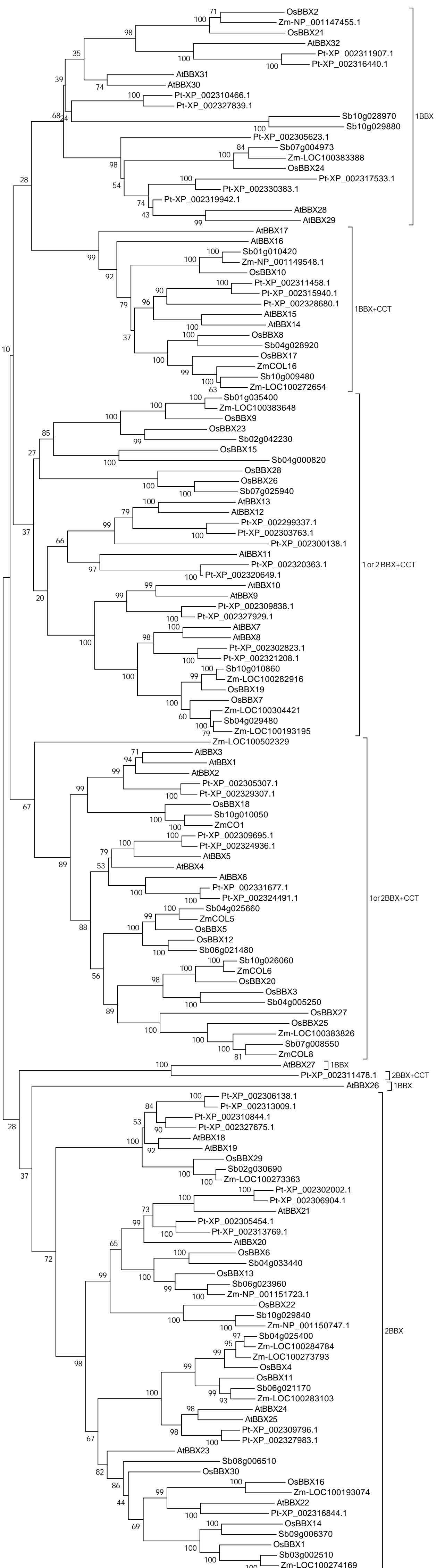

Supplement: Figure S4 — Phylogenetic analysis of the B-box TFs from rice, Arabidopsis , maize, poplar and sorghum bicolor . The sequences of the maize (Zm: Zea Mays), poplar ( Pt: Populus trichocarpa) and sorghum bicolor (Sb: Sorghum bicolor) BBX proteins were identified and downloaded from NCBI. The unrooted tree was generated from the full length protein sequences. The bootstrap values from 1000 replicates are indicated at each node. The scale bar represents 0.1 amino acid substitutions per site. (PDF) [file pone.0048242.s004.pdf]
