# Supplementary material for: The Rice B-Box Zinc Finger Gene Family: Genomic Identification, Characterization, Expression Profiling and Diurnal Analysis
Source: PLoS One. 2012 Oct 31;7(10):e48242. doi: 10.1371/journal.pone.0048242 (PMC3485221; doi:10.1371/journal.pone.0048242)
Supplement: Table S1 — Detailed information of the rice samples used in the microarray analysis. (DOC) [file pone.0048242.s005.doc]

**Table S1. Detailed information of rice samples used in microarray analysis**

| Sample No. | Developmental stage | Sample | Abbreviation |
| --- | --- | --- | --- |
| 1 | 72 hours after dry seed absorbed water | Seed | S |
| 2 | 3 days after sowing | Seedling 1 | Sl1 |
| 3 | Trefoil stage | Seedling 2 | Sl2 |
| 4 | Seedling with 2 tillers | Shoot | Sh |
| 5 | Seedling with 2 tillers | Root | R |
| 6 | Young panicle: secondary branch primordium differentiation | Leaf 1 | L1 |
| 7 | Young panicle: meiosis stage | Leaf 2 | L2 |
| 8 | Young panicle: secondary branch primordium differentiation | Sheath 1 | She1 |
| 9 | Young panicle: meiosis stage | Sheath 2 | She2 |
| 10 | 5 days before heading | Stem 1 | Ste1 |
| 11 | Heading stage | Stem 2 | Ste2 |
| 12 | 5 days before heading | Flag Leaf 1 | FL1 |
| 13 | 14 days after flowering | Flag Leaf 2 | FL2 |
| 14 | Young panicle: secondary branch primordium differentiation | Panicle 1 | P1 |
| 15 | Young panicle: pistil/stamen primordium differentiation | Panicle 2 | P2 |
| 16 | Young panicle: pollen-mother cell formation | Panicle 3 | P3 |
| 17 | Young panicle: meiosis stage | Panicle 4 | P4 |
| 18 | Heading stage | Panicle 5 | P5 |
| 19 | 1 day before flowering | Hull | H |
| 20 | 1 day before flowering | Stamen | Sta |
| 21 | 3 day after flowering | Spikelet | Spi |
| 22 | 7 days after pollination | Endosperm 1 | E1 |
| 23 | 14 days after pollination | Endosperm 2 | E2 |
| 24 | 21 days after pollination | Endosperm 3 | E3 |
