# Supplementary material for: The Rice B-Box Zinc Finger Gene Family: Genomic Identification, Characterization, Expression Profiling and Diurnal Analysis
Source: PLoS One. 2012 Oct 31;7(10):e48242. doi: 10.1371/journal.pone.0048242 (PMC3485221; doi:10.1371/journal.pone.0048242)
Supplement: Table S2 — Primers used for the qPCR analysis. (DOC) [file pone.0048242.s006.doc]

**Table S2.** Primers used for qPCR analysis.

| Gene | Primer name | Froward primer (5’-3’) | Reverse primer (5’-3’) |
| --- | --- | --- | --- |
| *OsBBX4* | *OsBBX4-*F/R | CGAGCATCATCACCATCATC | GTCGGAGAACTGGAGGAG |
| *OsBBX5* | *OsBBX5-*F/R | GAGGAGGATGCGGATGAT | CTCGTGGCTATTGTCGTC |
| *OsBBX6* | *OsBBX6-F/R* | CAGCAGCATCTCCGAGTA | CCTCCGATCCGTGTTACC |
| *OsBBX7* | *OsBBX7*-F/R | TGTTCAATGATGGGAGCGTA | TTGCTACATTCTGGCTGCAC |
| *OsBBX8* | *OsBBX8-F/R* | GCCGATTCGCAGTTCTTC | GAGTAATGGTGGTCGTTGG |
| *OsBBX9* | *OsBBX9-F/R* | GAACAACAACACAACACAACAC | TGCTGCTACCATTGCTCAT |
| *OsBBX10* | *OsBBX10*-F/R | ACGTGAAACTGGACCTGGAC | CGCTTCCTCTTCCTCTCCTC |
| *OsBBX11* | *OsBBX11*-F/R | TTGGGTTCAAGGATCTGGAG | CTCGTCGTCATCGGGTATCT |
| *OsBBX12* | *OsBBX12*-F/R | ACTCCGACCCTTACCTCGAC | CTCCGAGGATGATACGCTGT |
| *OsBBX13* | *OsBBX13*-F/R | GGAGGAAGAGGAGGAGGAA | AAATAGCCCGTCTGAGGAG |
| *OsBBX16* | *OsBBX16*-F/R | AGGTGTTCAAGTTGGCCTTG | TTGCTGAATCCACTGAACCA |
| *OsBBX17* | *OsBBX17-F/R* | GATGCTGGACATCGACTTC | TCGTAGTTGAGGCTGAGG |
| *OsBBX18* | *OsBBX18-F/R* | AGCAGCAGCATAGTGGTTA | CAGCAGGTGTCAGGATTCT |
| *OsBBX19* | *OsBBX19*-F/R | GCTGATTCAGGGATGTCGAT | GCACTGCTCTCACCAGTCAA |
| *OsBBX20* | *OsBBX20*-F/R | TCGACCCTTACCTCAACGTC | GGAGCTGTAGGACTGCTTGG |
| *OsBBX23* | *OsBBX23-F/R* | GCAAGCAAGACGGACATC | GCGGCAAATCTCTGAAATCT |
| *OsBBX26* | *OsBBX26*-F/R | TCAGAGGCCATGTACCATCA | CAGCTTCCTCGACTCGTACC |
| *OsBBX27* | *OsBBX27-F/R* | ACATCACCACCTCCTACTTG | GCCTCGTCTTCCTCTTCTC |
| *OsBBX29* | *OsBBX29-F/R* | GCCTTAGTTGTGATATGACTGTTC | GGTTTGCCATTTGCTCCTT |
| *OsBBX30* | *OsBBX30-F/R* | AGGAGAAGACGGGCTACT | CGATGCTGCTGCTTGTTG |
| *Ubiquitin* | Ubi-F/R | GGCTGACTACAACATCCA | GTGACCACACTTCTTCTTG |
